# Bacterial Effector Binding to Ribosomal Protein S3 Subverts NF-κB Function

**DOI:** 10.1371/journal.ppat.1000708

**Published:** 2009-12-24

**Authors:** Xiaofei Gao, Fengyi Wan, Kristina Mateo, Eduardo Callegari, Dan Wang, Wanyin Deng, Jose Puente, Feng Li, Michael S. Chaussee, B. Brett Finlay, Michael J. Lenardo, Philip R. Hardwidge

**Affiliations:** 1 Department of Microbiology, Molecular Genetics, and Immunology, University of Kansas Medical Center, Kansas City, Kansas, United States of America; 2 Laboratory of Immunology, National Institutes of Allergy and Infectious Diseases, Bethesda, Maryland, United States of America; 3 Department of Veterinary Science, South Dakota State University, Brookings, South Dakota, United States of America; 4 Division of Basic Biomedical Sciences, Sanford School of Medicine, University of South Dakota, Vermillion, South Dakota, United States of America; 5 Michael Smith Laboratories, University of British Columbia, Vancouver, British Columbia, Canada; 6 Departamento de Microbiología Molecular, Universidad Nacional Autónoma de México, Cuernavaca, Mexico; Duke University Medical Center, United States of America

## Abstract

Enteric bacterial pathogens cause food borne disease, which constitutes an enormous economic and health burden. Enterohemorrhagic *Escherichia coli* (EHEC) causes a severe bloody diarrhea following transmission to humans through various means, including contaminated beef and vegetable products, water, or through contact with animals. EHEC also causes a potentially fatal kidney disease (hemolytic uremic syndrome) for which there is no effective treatment or prophylaxis. EHEC and other enteric pathogens (e.g., enteropathogenic *E. coli* (EPEC), *Salmonella*, *Shigella*, *Yersinia*) utilize a type III secretion system (T3SS) to inject virulence proteins (effectors) into host cells. While it is known that T3SS effectors subvert host cell function to promote diarrheal disease and bacterial transmission, in many cases, the mechanisms by which these effectors bind to host proteins and disrupt the normal function of intestinal epithelial cells have not been completely characterized. In this study, we present evidence that the *E. coli* O157:H7 *nleH1* and *nleH2* genes encode T3SS effectors that bind to the human ribosomal protein S3 (RPS3), a subunit of nuclear factor kappa-light-chain-enhancer of activated B cells (NF-κB) transcriptional complexes. NleH1 and NleH2 co-localized with RPS3 in the cytoplasm, but not in cell nuclei. The N-terminal region of both NleH1 and NleH2 was required for binding to the N-terminus of RPS3. NleH1 and NleH2 are autophosphorylated Ser/Thr protein kinases, but their binding to RPS3 is independent of kinase activity. NleH1, but not NleH2, reduced the nuclear abundance of RPS3 without altering the p50 or p65 NF-κB subunits or affecting the phosphorylation state or abundance of the inhibitory NF-κB chaperone IκBα NleH1 repressed the transcription of a RPS3/NF-κB-dependent reporter plasmid, but did not inhibit the transcription of RPS3-independent reporters. In contrast, NleH2 stimulated RPS3-dependent transcription, as well as an AP-1-dependent reporter. We identified a region of NleH1 (N40-K45) that is at least partially responsible for the inhibitory activity of NleH1 toward RPS3. Deleting *nleH1* from *E. coli* O157:H7 produced a hypervirulent phenotype in a gnotobiotic piglet model of Shiga toxin-producing *E. coli* infection. We suggest that NleH may disrupt host innate immune responses by binding to a cofactor of host transcriptional complexes.

## Author Summary

The nuclear factor kappa-light-chain-enhancer of activated B cells (NF-κB) transcription factor complex plays an essential role in regulating the immune response to infection. Bacterial pathogens inject effector proteins into host cells to modulate the host innate immune response by interfering with NF-κB activation. We have discovered that *Escherichia coli* O157:H7, an important cause of hemorrhagic colitis, injects into mammalian cells an effector protein termed NleH1, which inhibits NF-κB-dependent transcription through a novel mechanism. NleH1 binds directly to a subunit of NF-κB, the ribosomal protein S3 (RPS3). One of the functions of RPS3 is to guide the recruitment of the p65 NF-κB subunit to specific promoters in response to different stimuli. NleH1, but not a closely related effector, NleH2, functions by reducing the nuclear abundance of RPS3 to dampen host transcriptional outputs. Our findings highlight a previously unappreciated mechanism by which bacterial effector proteins are able to alter host cell functions.

## Introduction

Diarrheal disease caused by enteric bacteria is an important endemic health threat and a major source of food borne disease [1]. Over 76 million cases of diarrheal disease and 5,000 deaths result from food borne illness in the United States annually (Centers for Disease Control and Prevention). Diarrheagenic strains of *E. coli* contribute greatly to the enormous economic and health burden of food borne disease. Enteropathogenic *E. coli* (EPEC) is a frequent cause of infantile diarrhea, while enterohemorrhagic *E. coli* (EHEC) has emerged as an important cause of hemorrhagic colitis in developed countries [2]. Often transmitted to humans through consumption of fruit juice, raw/undercooked meat, and vegetables contaminated with manure, EHEC is especially important because it is the leading cause of pediatric renal failure (hemolytic uremic syndrome; HUS).

EHEC adheres to intestinal enterocytes and produces a characteristic attaching/effacing (A/E) lesion resulting from localized intestinal microvilli destruction and the formation of a pedestal-like projection composed of epithelial-derived cytoskeletal components [3]. EHEC virulence proteins (effectors) are translocated directly into intestinal epithelial cells through a type III secretion system (T3SS; [4]). The T3SS is a molecular syringe, widely conserved among animal and plant pathogens, that directs the active transport of effectors into host cells. The EHEC T3SS and several effectors are encoded on a pathogenicity island termed the ‘locus of enterocyte effacement’ (LEE; [5]), whose genomic structure, function, and regulation are well conserved among the characterized A/E pathogens (i.e. EHEC, EPEC, *Citrobacter rodentium*). Over 20 other secreted proteins (non-LEE-encoded effectors; Nles) encoded by genes in multiple pathogenicity islands located throughout the EHEC genome have also been described [6]. A bioinformatics study recently employed homology searches against other bacterial effectors to identify 39 secreted/translocated effectors encoded in the EHEC Sakai genome [7]. Several studies have reported the importance of Nles to virulence [6],[8],[9],[10],[11],[12],[13],[14].

Intestinal epithelial cells have evolved mechanisms to prevent infection by pathogens by inhibiting bacterial colonization and by interacting with the underlying immune system [15]. Paneth cells located at the base of intestinal crypts produce proinflammatory cytokines and express Toll-like receptors (TLRs) and nucleotide-binding oligomerization domain (NOD) proteins. NOD proteins recognize pathogen-associated molecular patterns (PAMPs) and promote activation of host proinflammatory signaling pathways [16], many of which are regulated by the nuclear factor kappa-light-chain-enhancer of activated B cells (NF-κB). PAMP detection typically initiates a signal transduction cascade that promotes polyubiquitination of the inhibitor of NF-κB (IκB)-kinase-γ complex (NEMO), ultimately leading to NF-κB activation through the degradation of the inhibitory NF-κB chaperone IκBα. IκBα masks the nuclear localization signal (NLS) of p65, yet leaves the p50 nuclear localization signal exposed, permitting flux of IκBα/NF-κB complexes between the nucleus and the cytoplasm [17]. IκBα degradation shifts the balance to favor increased nuclear localization of NF-κB.

NF-κB homo- and hetero-dimers (typically composed of the p50 and p65 subunits) bind to DNA (κB sites) within target gene promoters and regulate transcription by recruiting co-activator/repressor molecules [18]. It is not completely clear how diverse stimuli generate unique transcriptional responses in different cells and tissues [19]. However, the recent discovery of a non-Rel NF-κB subunit, RPS3, which guides NF-κB to specific κB sites after specific cellular stimuli, has shed some light on this issue. RPS3 was detected as a major co-purifying molecule with the p65 NF-κB subunit [18]. The N-terminal region of RPS3, notably the KH domain, is required for binding to the N-terminal portion of p65 [18]. RPS3 forms a complex with p65 that dramatically increases the affinity of NF-κB complexes for a subset of target genes and provides a mechanism by which selected promoters could be activated in response to specific stimuli [18].

The identity and underlying mechanism of action of *E. coli* PAMPs and effector proteins that respectively stimulate vs. repress host innate immunity have been incompletely characterized. *E. coli* flagellin induces secretion of interleukin (IL)-8 from intestinal epithelial cells [20]. However, IL-8 secretion is inhibited relatively early during infection by the delivery of one or more unidentified T3SS effectors [20],[21]. These effectors are believed to function by preventing the degradation of IκBα, to reduce NF-κB translocation into the nucleus [20]. That the nuclear abundance of NF-κB and its affinity for DNA are increased at early stages of infection, without concomitant increases in IL-8 expression [20],[22],[23], may also suggest effector-mediated inhibition of innate responses. Despite significant effort, how T3SS effectors coordinate their activities to suppress the potent host inflammatory response normally induced by flagellin and other PAMPs remains incompletely characterized.

*E. coli* O157:H7 EDL933 contains two copies of the *nleH* gene, designated *nleH1* (Z0989) and *nleH2* (Z6021). Each gene encodes predicted protein products with significant sequence similarity to *Shigella* OspG [7],[24],[25], a protein known to interfere with NF-κB activation [26]. *C. rodentium*, a pathogen of mice that shares many pathogenic strategies with EHEC, rapidly induces the nuclear translocation of NF-κB in host cells [27]. *C. rodentium* NleH has been shown to play an important role in the colonization of animal hosts and in altering NF-κB activity [25]. We therefore undertook a biochemical analysis of the host binding partners and mechanism of action of the *E. coli* O157:H7 effectors NleH1 and NleH2 to understand better how bacterial effectors modulate host innate immunity. In this study we show that the *E. coli* O157:H7 NleH proteins play an important role in host-pathogen interactions by binding to the human ribosomal protein S3 (RPS3), a newly identified subunit of NF-κB [18]. Our results suggest a potentially novel mechanism for bacterial effector-mediated disruption of host innate responses to infection.

## Results

### NleH1 and NleH2 are injected into host cells by the T3SS

Recent proteomic [6] and bioinformatic [7] screens identified a repertoire of novel T3SS-effectors encoded on pathogenicity islands throughout EHEC genomes in non-locus of enterocyte effacement pathogenicity islands. Among these, *E. coli* O157:H7 strain EDL933 contains two copies of the *nleH* gene, designated *nleH1* (Z0989) and *nleH2* (Z6021), which are predicted to encode T3SS substrates [6]. NleH1 (293 amino acids) and NleH2 (303 amino acids) are 84% identical and encoded on distinct, non-LEE pathogenicity islands (*O*-islands 36 and 71, respectively) from which other T3SS-effectors with proven roles in bacterial virulence (e.g. NleA, NleD) are expressed [11],[14]. Others have noted NleH sequence similarity to *Shigella flexneri* OspG and serine/threonine protein kinases of *Yersinia* spp. [7].

Both NleH1 and NleH2 contain a lysine residue (K159 and K169, respectively) present in a Ser/Thr protein kinase domain that based on homology to *Shigella* OspG, may function as the catalytic site of kinase activity. Despite 84% shared identity, NleH1 differs from NleH2 in that NleH1 lacks a 10 amino acid insertion containing putative SH2 and PKA-interaction domains (Figure S1A). NleH1 also possesses several potential Ser/Thr phosphorylation sites lacking in NleH2.

Other A/E pathogens also encode multiple copies of *nleH*. The EHEC Sakai strain encodes *nleH1* and *nleH2* [7]. The recently sequenced EPEC 2348/69 encodes three copies of *nleH*, one of which may be a pseudogene [28]. *nleH* genes are present in other EPEC genomes, including B171 [28] and E22 [29]. NleH1 and NleH2 are also 83% identical to NleH encoded by *C. rodentium* [25]. Only one copy of NleH is present in *C. rodentium* [24]. This copy of NleH appears to be more similar to EHEC NleH1 than to NleH2, as it lacks the 10 amino-acid insertion that is present in EHEC NleH2, but absent in NleH1 [24].

To determine if EHEC NleH1 and/or NleH2 are translocated into host cells by the *E. coli* T3SS, we constructed fusions to the TEM-1 β-lactamase. This reporter system has proven robust for assaying effector translocation into host cells [30]. These constructs were introduced into both wild type and Δ*escN* EPEC, a strain deficient in T3SS function that is commonly used to evaluate the dependence of A/E pathogen effector translocation on the T3SS. These strains were used to infect HeLa cells loaded with the CCF2/AM substrate. We used a fluorescence microplate reader to quantify b-lactamase activity in host cells after a 4 h infection. While NleH1- and NleH2-TEM were expressed at similar levels in both wild type and Δ*escN* EPEC (Figure S1B), the proteins were detectably translocated into mammalian cells only by wild type (p<0.001, ANOVA), indicating, as predicted from studies of *C. rodentium* NleH [24], that both EHEC NleH1 and NleH2 are translocated into host cells by the T3SS (Figure S1C).

**Figure 1:**
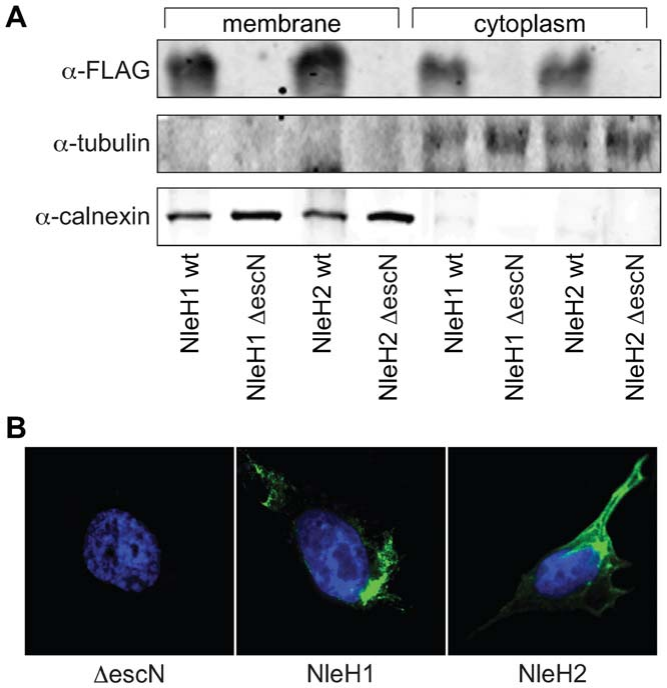
Translocation and localization of NleH1 and NleH2 in host cells. **A.** Immunoblot analysis of cytoplasmic and membrane HeLa cell fractions following infection with EHEC strains expressing NleH1- or NleH2-FLAG fusions. Blots were probed with α-FLAG, α-tubulin, and α-calnexin antibodies. **B.** Immunofluorescence microscopy analysis of NleH localization. HeLa cells were infected with EHEC Δ*escN* (left), EHEC/p*nleH1*-FLAG (middle), or EHEC/p*nleH2*-FLAG (right) and stained with DAPI (blue) and an α-FLAG monoclonal antibody (green).

To validate these data and begin to determine the subcellular localization of NleH1 and NleH2, we constructed fusions to the FLAG epitope in pFLAG-CTC, expressed these plasmids in EHEC, and infected HeLa cells. After 4 h, we removed the extracellular bacteria and subjected the cell lysates to immunoblotting. The expected distribution of calnexin and tubulin validated the integrity of membrane and cytoplasmic fractions, respectively. NleH1 and NleH2 were detected in both the host cytoplasmic and membrane fractions (Figure 1A). We obtained similar data after we fractionated host cells infected with EPEC strains expressing FLAG fusions to NleH1 and NleH2 (Figure S1D). We also employed immunofluorescence microscopy to assess the intracellular localization of NleH1 and NleH2 by infecting HeLa cells with EHEC strains expressing either NleH1 or NleH2 fused to the FLAG epitope. NleH1 and NleH2 both localized to the cell periphery and around, but not in, the host nucleus (Figure 1B).

### NleH1 and NleH2 bind the human ribosomal protein S3 (RPS3)

To identify eukaryotic cell binding partners of NleH, we used purified His-NleH1 to isolate mammalian proteins that bind to this effector. We incubated His-NleH1 with HeLa cell lysates and captured His-NleH1 and any co-purifying host proteins through passage over Ni-NTA agarose resin (Figure S2A). Using mass spectrometry, we identified a protein with an apparent molecular mass of ∼28 kDa that selectively eluted with NleH1 in affinity purification experiments as RPS3, a nucleic acid-binding KH domain protein implicated in DNA repair [31], and the regulation of NF-κB-dependent transcription [18].

**Figure 2:**
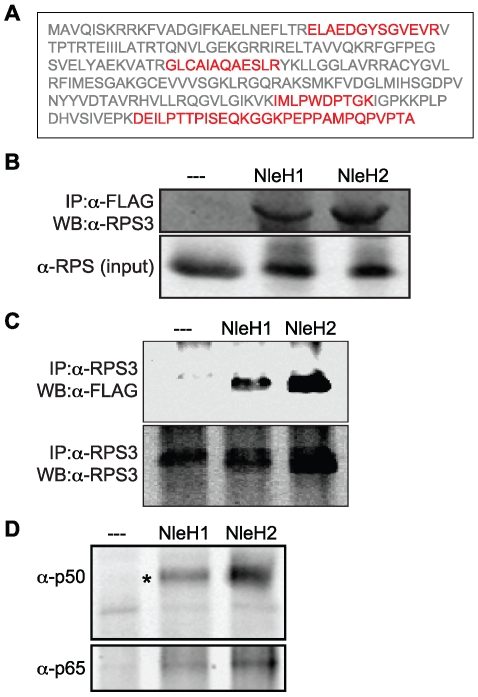
NleH1 and NleH2 bind to the host ribosomal protein S3 (RPS3). **A.** RPS3 amino acid sequence. Tryptic peptides identified by mass spectrometry are indicated in red. **B.** Immunoprecipitation of RPS3 from HeLa cells by translocated NleH1- and NleH2-FLAG following infection with EPEC UMD207. Samples were immunoprecipitated with α-FLAG antibody and immunoblotted for RPS3. The lower panel indicates the RPS3 abundance in the cell lysates. **C.** Immunoprecipitation of NleH1- and NleH2-FLAG from HeLa cells by RPS3 following infection with EPEC UMD207. Samples were immunoprecipitated with α-RPS3 antibody and immunoblotted for FLAG to detect NleH. The lower panel demonstrates the equal enrichment of RPS3 among samples following α-RPS3 immunoprecipitation. **D.** Enrichment of the p50 and p65 NF-κB subunits by NleH1 and NleH2. Samples were immunoprecipitated with α-FLAG antibody and immunoblotted for p50 (top) and p65 (bottom).

To verify that RPS3 interacts with NleH1 (Figure 2A), we used an a-RPS3 antibody to immunoprecipitate RPS3 from cell lysates obtained after infection with EPEC UMD207 [eae^-^/bfp^-^; [32]] strains expressing FLAG-tagged NleH1, NleH2, or a control FLAG epitope. We used this strain because it lacks both the intimin protein and the bundle-forming pilus (BFP), thus facilitating removal of the bacteria after infection, to reduce potential contamination of translocated, immunoprecipitated NleH with bacterial proteins. Both NleH1 and NleH2 co-immunoprecipitated RPS3, supporting the affinity purification data (Figure 2B). Likewise, immunoprecipitated RPS3 selectively enriched for translocated NleH1 and NleH2 (Figure 2C).

To provide additional evidence for the specificity of NleH-RPS3 binding, we assessed the ability of NleH1 to bind to a different ribosomal protein, RPS16. We determined that NleH1 did not significantly bind to RPS16 (Figure S2B). Similarly, we assessed the ability of a different EHEC effector protein, NleF [9], to interact with RPS3. We determined that NleF could not effectively immunoprecipitate RPS3 following its translocation into host cells (Figure S2C).

RPS3 binds directly to the p65 NF-κB subunit [18]. We therefore tested whether NleH associated with other NF-κB proteins through RPS3 interaction. Immunoprecipitation of NleH1 and NleH2 also enriched for the NF-κB subunits p50 and p65 (Figure 2D). We also performed experiments by depleting RPS3 from cell lysates using an a-RPS3 antibody. When these RPS3-depleted cell lysates were immunoprecipitated for NleH, we were no longer able to detect either p50 or p65 (data not shown), further substantiating that NleH interacts indirectly with NF-κB via binding to RPS3.

Because of the homology between NleH and *Shigella* OspG, a protein that binds ubiquitin-conjugating enzymes (E2s), including UbcH5 and UbcH7 [26], we also tested if NleH interacted with the host ubiquitin machinery. To do this we immunoprecipitated NleH1 from cell lysates and performed immunoblotting experiments to detect ubiquitination of NleH1 or its binding to Ub-associated proteins. We did not obtain evidence of such interactions (data not shown). Taken together, these biochemical data suggest that NleH1 and NleH2 possess a novel host substrate, RPS3, a protein that interacts with NF-κB to regulate mammalian transcription.

### BiFC analysis of NleH-RPS3 interaction

**Figure 3:**
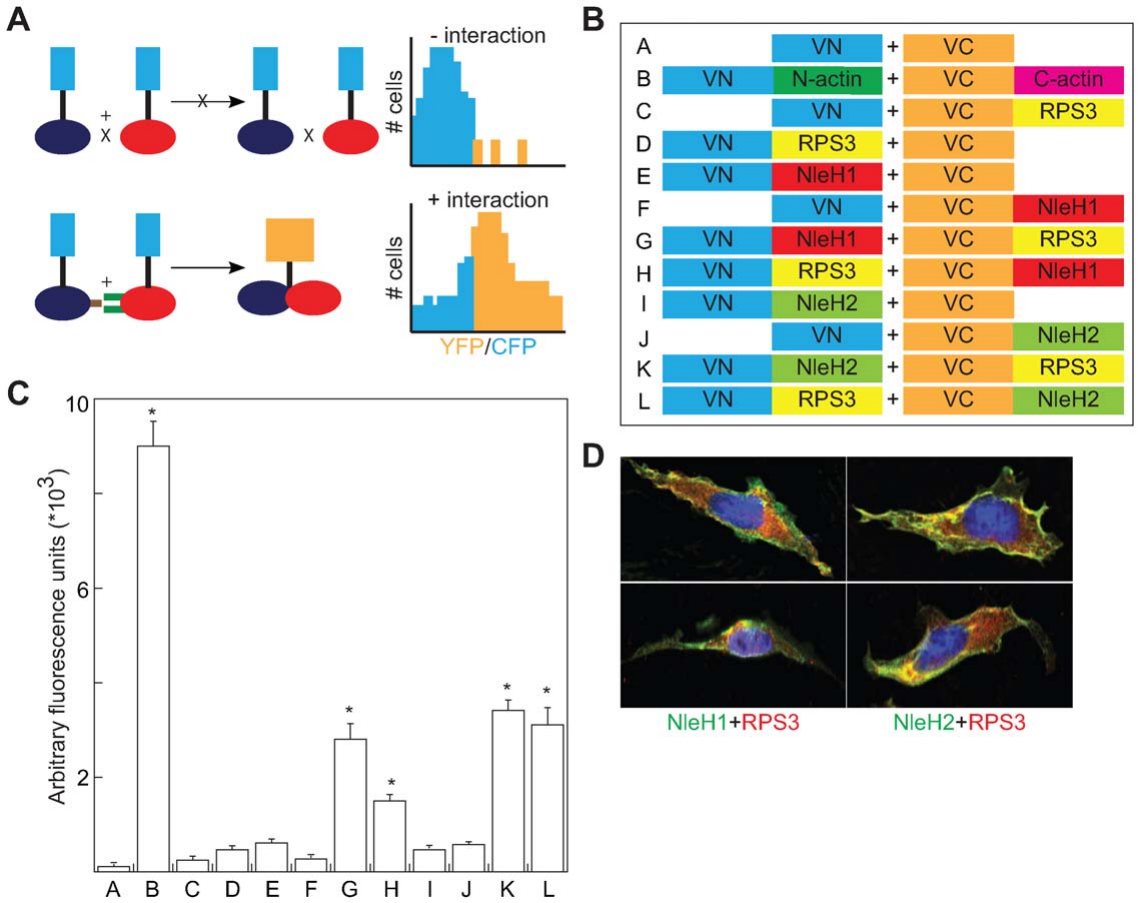
Bimolecular fluorescence complementation analysis of NleH-RPS3 interaction. **A.** BiFC schematic. Protein-protein interaction promotes the reconstitution of a functional fluorophore, measured as an increase in the YFP:CFP emission ratio [33],[34]. **B.** Experimental design of NleH- and RPS3-eYFP fusions. VN, N-terminus (AAs 1–173) of Venus fluorescence protein; VC, C-terminus (AAs 155–238) of Venus fluorescence protein. **C.** Relative fluorescence intensity resulting from the co-transfection of the indicated NleH- and RPS3-eYFP plasmid combinations (n = 3). Asterisks indicate significantly different fluorescence intensity compared with uninfected samples (p<0.05, ANOVA). **D.** Confocal immunofluorescence microscopy analysis of NleH and RPS3 co-localization. HeLa cells were infected with EPEC strains expressing NleH1- or NleH2-FLAG and stained with DAPI (blue), α-FLAG (green), and α-RPS3 (red) antibodies. Two representative cells are shown for each infection condition.

We next sought to confirm immunoprecipitation data with an additional protein interaction assay, bimolecular fluorescence complementation (BiFC). BiFC assays are based on the reconstitution of two fragments of the enhanced yellow fluorescent protein (eYFP) when they are brought in close proximity by an interaction between proteins fused to the YFP fragments (Figure 3A; [33],[34]). Protein-protein interactions generate a fluorescent signal at the site of the protein complex, permitting quantification or direct visualization of the interaction.

We based our design of NleH and RPS3 BiFC constructs on the BiFC assay originally developed by Hu et al. [35],[36]. This design utilizes Venus, an enhanced yellow fluorescent protein (eYFP) variant, which emits strong fluorescence [33] without requiring significant protein over-expression [37]. We generated protein chimeras with split N- and C-terminal fragments (VN and VC, respectively), of eYFP. Then we designed plasmids to contain a linker region between the eYFP fragment and a multi-cloning site. HA epitope tags were added to facilitate detection with immunoblotting. We also constructed fusions to actin, a protein often used as a positive control in BiFC assays, as well as fusions to RPS3 and NleH1 and NleH2 (Figure 3B). These constructs were transfected into HeLa cells and analyzed 48 h post-transfection.

The reconstitution of eYFP mediated by interaction between the N- and C-termini of actin generated intense fluorescence (Figure 3C). In contrast, the expression of individual constructs or constructs lacking both actin fragments did not generate significant fluorescence. We then examined the potential for interaction between RPS3 and NleH1 or NleH2. The co-expression of eYFP chimeras of RPS3 and both NleH1 and NleH2 also reconstituted YFP fluorescence (p<0.001, ANOVA), whereas transfection of individual plasmids did not (Figure 3C), suggesting that both NleH1 and NleH2 bind RPS3 in mammalian cells. Similar data were obtained irrespective of the position of the eYFP fusion relative to NleH or RPS3 (e.g. compare Figure 3C, lanes G–H and K–L). In addition, we used confocal immunofluorescence microscopy after infecting cells with EPEC strains expressing NleH1- or NleH2-FLAG to determine that both NleH1 and NleH2 co-localize with endogenous RPS3 in the cytoplasm (Figure 3D). These microscopy data support the previously described biochemical analyses that showed an interaction between NleH and RPS3.

### NleH N-termini bind to the N-terminus of RPS3

**Figure 4:**
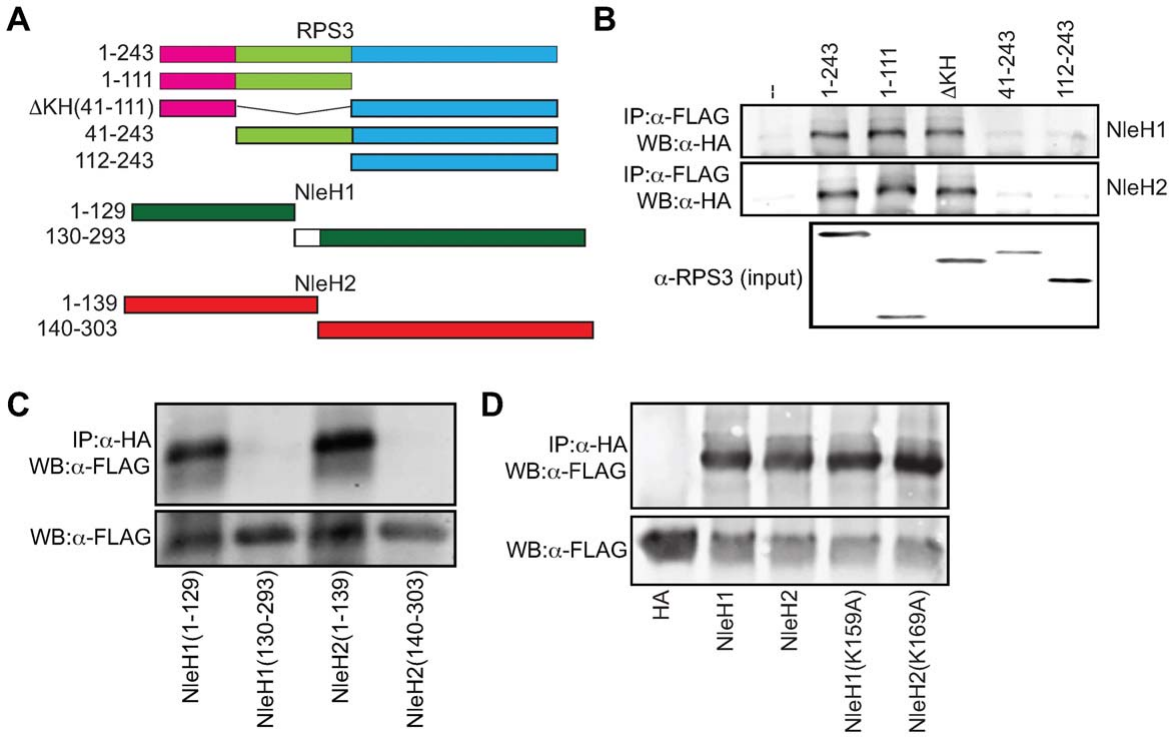
N-termini of NleH1 and NleH2 bind to the N-terminus of RPS3. **A.** Design of RPS3 and NleH protein truncations. The amino acids present in each truncation are indicated to the left of the figure. The death (pink), KH (green), and DNA-repair (blue) domains of RPS3 are indicated. The white box depicts the 10 amino acids lacking in NleH1 but present in NleH2. **B.** Co-immunoprecipitation of NleH1- (top) and NleH2-HA (middle) with RPS3-FLAG truncations. Samples were immunoprecipitated with α-FLAG antibody to capture RPS3 and immunoblotted for HA to detect NleH. The bottom panel depicts the expression levels of the RPS3-FLAG truncations. **C.** Co-immunoprecipitation of RPS3-FLAG with NleH1- and NleH2-HA truncations. Samples were immunoprecipitated with α-HA antibody to capture NleH and immunoblotted for FLAG to detect RPS3. The lower panel depicts the expression levels of the NleH truncations. **D.** Immunoprecipitation of RPS3-FLAG with NleH1(K159A)-HA and NleH2(K169A)-HA. Samples were immunoprecipitated with α-HA antibody to capture NleH and immunoblotted for FLAG to detect RPS3. The lower panel depicts the expression levels of RPS3 in cell lysates.

To map the binding domain of RPS3 on NleH1 and NleH2, we carried out a structure-function study with deletions of RPS3 that encompass the known functional domains of this protein (Figure 4A). Co-immunoprecipitation experiments revealed that the region of RPS3 (AAs 1–41) N-terminal to the KH domain is required for RPS3 binding to both NleH1 and NleH2 (Figure 4B). A similar analysis revealed that the N-termini of NleH1 and NleH2 (AAs 1–139 and 1–149, respectively) were required for binding to RPS3 (Figure 4C).

### NleH1 and NleH2 are autophosphorylated Ser/Thr protein kinases

Given the sequence conservation between NleH and a domain implicated in the autophosphorylation and kinase activities of *Shigella* OspG [7],[26], we examined if NleH1 and NleH2 are also Ser/Thr protein kinases. We purified His-NleH1 and NleH2, as well as NleH site-directed mutants in which a lysine residue was mutated to alanine [NleH1(K159A) and NleH2(K169A)]. By performing Pro-Q staining of purified proteins, we determined that, similar to OspG [26], both NleH1 and NleH2 are autophosphorylated (Figure S3A). We also used these proteins to assay for phosphorylation of the myelin basic protein (MBP), a commonly used substrate in protein phosphorylation assays. Both wild-type NleH1 and NleH2, but not the site directed mutants [NleH1(K159A) or NleH2(K169A)], phosphorylated MBP *in vitro* (Figure S3B), suggesting that NleH1 and NleH2 possess kinase activity.

RPS3 is phosphorylated by the extracellular signal-regulated kinase 1 (ERK1) on T42 [38]. This event is necessary for RPS3 nuclear translocation in response to DNA damage [39] and may play a role in the transcriptional activities of RPS3 [18]. The non-ribosomal fraction of RPS3 is also phosphorylated by PKCδ on S6 and T221 to promote its nuclear translocation [40]. To determine if NleH kinase activity is essential to RPS3 binding, we immunoprecipitated RPS3 and interrogated the samples for the presence of wild type or kinase-deficient NleH1/NleH2. We observed that NleH-RPS3 binding was independent of NleH kinase activity (Figure 4D).

To determine if NleH could phosphorylate RPS3, we conducted *in vitro* kinase assays with NleH and RPR3 and analyzed the results by immunoblotting with an α-phospho-Ser/Thr-specific antibody (the utility of which for RPS3 phosphorylation studies has been documented previously [40]), following separation by SDS-PAGE. We determined that while NleH1 and NleH2 could phosphorylate MBP, and ERK1 could phosphorylate RPS3, NleH1 and NleH2 had no detectable ability to phosphorylate RPS3 (data not shown). Overall, these data suggest that the NleH-RPS3 interaction is independent of NleH kinase activity. Further, they leave open the possibility that NleH effectors may be multifunctional proteins with the capacity to target multiple host regulatory pathways.

### NleH1 reduces the nuclear abundance of RPS3

RPS3 translocates to the nucleus after stimulation with tumor necrosis factor-alpha (TNF-α) [18]. NleH1 and NleH2 bind to the N-terminus of RPS3, potentially masking its putative nuclear localization signal. To determine if NleH binding to RPS3 inhibits translocation of RPS3 into the nucleus, we first used immunofluorescence microscopy to evaluate the relative abundance of nuclear vs. cytoplasmic RPS3. The percentage of cells containing predominantly nuclear RPS3 was significantly reduced in cells infected with wild type (8±3%) vs. Δ*escN* EHEC (36±10%; p = 0.01, t-test), suggesting an EHEC T3SS effector alters RPS3 nuclear abundance (Figure S4A-B).

**Figure 5:**
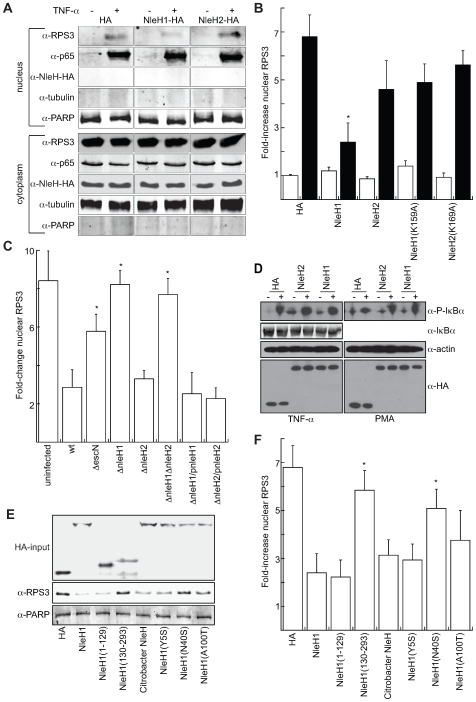
NleH1 reduces the nuclear abundance of RPS3. **A.** Immunoblot analysis of cytoplasmic and nuclear fractions of 293T cells transfected with NleH1, NleH2, or an HA-epitope control, in the presence or absence of TNF-α (100 ng/ml) stimulation for 1 h. Blots were probed with α-RPS3, α-p65, α-HA, α-tubulin, and α-PARP monoclonal antibodies. **B.** Quantification (n≥4) of the fold-increase in nuclear RPS3 as assessed by densitometry analysis of immunoblots in the absence (open bars) or presence (black bars) of TNF-α stimulation. RPS3 signal intensity was normalized to tubulin (cytoplasmic) and PARP (nuclear). Asterisks indicate significantly different compared with HA transfection (p<0.05, ANOVA). **C.** Quantification of the fold-increase in nuclear RPS3 following a 3 h infection of HeLa cells with *E. coli* O157:H7 EDL933 strains possessing or lacking *nleH1* and/or *nleH2*, as well as with strains complemented with the indicated NleH plasmids (n = 3). Asterisks indicate significantly different compared with wild-type infection (p<0.05, ANOVA). **D.** Immunoblot analysis of IκBα phosphorylation induced by TNF-α (left) or PMA (right), and total IκBα, in the presence or absence of NleH. **E.** Immunoblot analysis of the impact of *C. rodentium* NleH and EHEC NleH1 truncations and site-directed mutants on RPS3 nuclear abundance. Blots were probed with α-HA (top), α-RPS (middle), and α-PARP (bottom) monoclonal antibodies. The HA-input panel depicts the expression levels of the indicated constructs. The α-RPS panel depicts the nuclear abundance of RPS3 after stimulation with TNF-α in 293T cells transfected with the indicated constructs. The α-PARP signal was used for normalization of immunoblot signal intensities. **F.** Quantification (n = 3) of the fold-increase in nuclear RPS3 as assessed by immunoblotting in the presence of the indicated NleH expression plasmids. Asterisks indicate significantly different compared with NleH1 transfection (p<0.05, ANOVA).

We evaluated the relative abundance of nuclear vs. cytoplasmic RPS3 in 293T cells in the presence or absence of transfected NleH1- and NleH2-HA with or without 1 h stimulation with 100 ng/µl TNF-α. We subsequently fractionated the cells to separate nuclear from cytoplasmic components. We quantified poly(ADP-ribose) polymerase (PARP) and tubulin abundance to normalize the concentrations of nuclear and cytoplasmic subcellular protein fractions. The lack of PARP in the cytoplasmic fraction and the lack of tubulin in the nuclear fraction demonstrated the absence of cross-contamination between the fractions (Figure 5A). As expected from previous reports utilizing similar treatment conditions [18], the nuclear abundance of both RPS3 and p65 significantly increased after stimulation with TNF-α. Most notably, the nuclear abundance of RPS3 was significantly reduced in samples containing NleH1, while nuclear p65 and cytoplasmic concentrations of both RPS3 and p65 were unchanged. NleH1 and NleH2 were expressed equally among all samples, but were detected only in the cytoplasm, rather than the nuclear fraction (Figure 5A), consistent with immunofluorescence microscopy data.

Quantitative analysis of RPS3 abundance (based on densitometry analysis of immunoblots) revealed that NleH1 significantly reduced (∼65%) the relative abundance of nuclear RPS3 (Figure 5B, p<0.001, ANOVA) after stimulation with TNF-α. In contrast, NleH2 transfection did not significantly alter RPS3 nuclear abundance. Neither protein altered the nuclear abundance of the NF-κB p65 subunit (Figure S5A). The nuclear abundance of p50 was also unchanged as a function of NleH expression (data not shown), indicating that NleH1 activity is specific to RPS3. The kinase activity of NleH1 was important for its ability to reduce RPS3 nuclear abundance, as the effect of transfecting NleH1(K159A) was not significantly different from that of transfecting the HA control plasmid (Figure 5B).

Immunofluorescence microscopy analyses support these biochemical data. We observed that RPS3 translocated into the nuclei of HeLa cells after TNF-α stimulation. The nuclear translocation of RPS3 was inhibited in cells infected with EPEC expressing NleH1-FLAG, but not NleH2-FLAG, relative to uninfected controls (Figure S5B). We also used an α-p65 antibody for immunoprecipitation of nuclear extracts to validate further that the impact of NleH1 on reducing RPS3 nuclear abundance would alter the nuclear association of RPS3 with p65. Our analysis of immunoprecipitated nuclear p65 samples indicated a reduced abundance of RPS3 in samples co-transfected with NleH1, but not NleH2, relative to an HA epitope control (Figure S5C), also suggesting that NleH1 could interfere with RPS3-p65 interactions by reducing RPS3 nuclear abundance.

To test further the hypothesis that NleH1 reduces RPS3 nuclear abundance, we also quantified changes in nuclear RPS3 after infecting HeLa cells with EHEC strains possessing or lacking *nleH1* and/or *nleH2* constructed using lambda Red mutagenesis [41]. After 3 h of infection, bacteria were killed with antibiotics and TNF-α was added at 100 ng/ml for 1 h to stimulate RPS3 nuclear translocation. Infection with Δ*nleH1* EHEC failed to inhibit RPS3 nuclear translocation, whereas infection with Δ*nleH2* was not significantly different from wild type EHEC (Figure 5C). Our analysis of the Δ*nleH1*Δ*nleH2* mutant also indicated that NleH1 is the EHEC effector primarily responsible for reducing the nuclear abundance of RPS3. Complementing *nleH* mutants with corresponding NleH-FLAG plasmids confirmed the dependence of alterations in RPS3 nuclear abundance upon NleH1 (Figure 5C). Neither NleH1 nor NleH2 impaired the TNF-α or phorbol 12-myristate 13-acetate (PMA)-induced phosphorylation of IkBa, nor did they alter total IκBα concentrations (Figure 5D), suggesting that unlike OspG [26], NleH1 and NleH2 do not alter the degradation of the inhibitory IκBα subunit. Overall, these data support a role for NleH1, but not NleH2, in the reduction of RPS3 nuclear abundance, without significant impact on other NF-κB subunits. While the NleH1 K159 residue was non-essential for RPS3 binding, it was important to the ability of NleH1 to reduce RPS3 nuclear abundance, suggesting that other host proteins or translocated effectors may be involved.

### Functional differences between NleH1 and NleH2

Because our binding studies suggested that both NleH1 and NleH2 bind to RPS3, we were somewhat surprised to observe that only NleH1, and not NleH2, reduced RPS3 nuclear abundance. To begin to elucidate the functional differences between these two effectors, we sought to generate and characterize mutations in NleH1 that would still be competent for RPS3 binding, yet would fail to alter RPS3 nuclear abundance. Because we observed that NleH binding to RPS3 is mediated by the N-terminus, we targeted this region for mutagenesis and focused on several amino acids that differ between NleH1 and NleH2.

We first confirmed that *C. rodentium* NleH, which appears to be more similar to EHEC NleH1 than NleH2, also reduced RPS3 nuclear abundance (Figure 5E–F). By transfecting individually the N- (AAs 1–129) and C-terminal (AAs 130–293) regions of NleH1, we also determined that the N-terminus of NleH1 was sufficient to reduce RPS3 nuclear abundance. We then constructed NleH1 mutants in which residues of NleH1 were swapped for those appearing in NleH2. These point mutants (Y5S; N40S,A42T,K45T; and A100T,A101T,M102I,I106S) were expressed at similar levels as wild type NleH1 and were able to bind RPS3 (Figure 5E and S5D). We determined that mutating the N40,A42,K45 region of NleH1 to S40,T42,T45 was sufficient to prevent the NleH1-mediated inhibition of RPS3 nuclear abundance (Figure 5E–F). Mutating the A100,A101,M102,I106 region had a modest, but statistically insignificant influence on NleH1 activity, whereas we found that mutating Y5 had no impact on NleH1 activity. Since *C. rodentium* NleH appears to have similar activity to EHEC NleH1, modulating RPS3 dynamics may be a strategy employed by multiple A/E pathogens.

### NleH1 and NleH2 differentially regulate NF-kB-dependent transcriptional activity

After translocating into the nucleus, RPS3 selectively alters the expression of a subset of kB-dependent gene promoters to generate specific transcriptional responses to diverse extracellular signals [18]. p65 binding to κB sites in IκBα, IL-8, and IL-2 promoters is significantly reduced in RPS3-knockdown cells [18], whereas RPS3-independent genes (e.g. CD25 and CD69) are unaffected. This observation suggests that selective gene expression is due to differing requirements of specific promoter sites for the RPS3 subunit of NF-κB. Because NleH1 and NleH2 bind to RPS3, with NleH1 reducing its nuclear abundance, and because RPS3 regulates NF-κB activity, we hypothesized that NleH1 and/or NleH2 might also alter NF-κB dependent transcription.

To test this hypothesis, we co-transfected HeLa cells with a firefly luciferase construct driven by a consensus κB site previously demonstrated to be responsive to RPS3 [18], to measure NF-κB activity. For data normalization, we used a renilla luciferase plasmid. We quantified luciferase activity and then calculated the fold-induction of NF-κB activity relative to unstimulated or uninfected cells.

**Figure 6:**
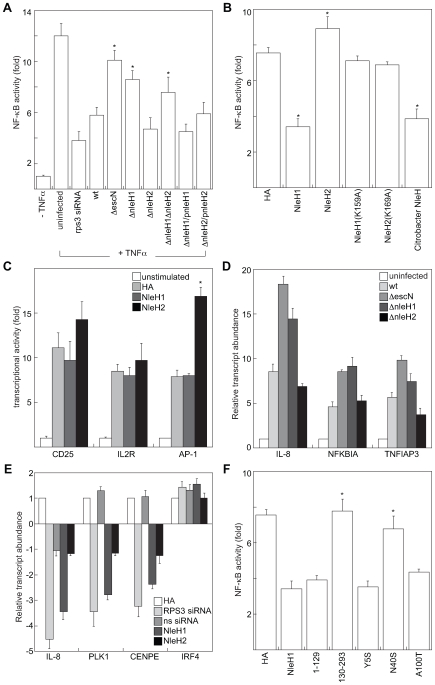
NleH effectors alter host NF-κB activity. **A.** Relative NF-κB activity (compared with uninfected cells) as a function of TNF-α stimulation, siRNA transfection, and/or infection with EHEC strains possessing or lacking *nleH1* and/or *nleH2* (n≥4). HeLa cells were co-transfected with a firefly luciferase construct driven by a consensus κB site and a renilla luciferase plasmid, cultured for 36 h, and then infected with EHEC strains for 3 h in the presence or absence of TNF-α stimulation or silencing with rps3 siRNA. Asterisks indicate significantly different compared with wild-type infection (p<0.05, ANOVA). **B.** Relative NF-κB activity in 293T cells transfected with the indicated NleH plasmids (n = 4). After 36 h, cells were stimulated with TNF-α (100 ng/ml, 1 h). Asterisks indicate significantly different compared with HA transfection (p<0.05, ANOVA). **C.** Impact of NleH1 and NleH2 on CD25 (left), IL-2R (middle), and AP-1 (right)-dependent luciferase reporter activity. 293T cells were transfected with the indicated reporter plasmids and treated with either TNF-α (CD-25 and IL-2R) or PMA (AP-1) 36 h post-transfection (n = 3). Asterisks indicate significantly different compared with HA transfection (p<0.05, ANOVA). **D.** Relative transcript abundance, relative to uninfected cells assessed by RT-PCR analysis of 293T cells infected for 4 h with the indicated bacterial strains. IL-8, NFKBIA, and TNFIAP3 data were normalized to GAPDH expression. **E.** Relative transcript abundance in 293T cells after 48 h transfection with HA, NleH1-HA, NleH2-HA, and RPS3-specific or sequence-scrambled (ns) siRNA constructs. IL-8, PLK1, CENPE, and IRF4 data were normalized to GAPDH expression. **F.** Impact of *C. rodentium* NleH and EHEC NleH1 truncations and site-directed mutants on RPS3/NF-κB-dependent transcriptional activity. Experiments were performed as described in panel B, using the plasmids indicated on the x-axis (n = 3). Asterisks indicate significantly different compared with NleH1 transfection (p<0.05, ANOVA).

Treating cells with TNF-α stimulated NF-κB-dependent luciferase activity ∼12-fold (Figure 6A). Transfecting RPS3 siRNA reduced RPS3 protein abundance to ∼20% of native levels (Figure S6A) and reduced the effect of TNF-α stimulation ∼4-fold (Figures 6A and S6B), confirming the sensitivity of the luciferase assay and importantly, the significant dependence upon RPS3 for efficient NF-κB transcription from this κB site ([18]; Figure 6A). We infected HeLa cells with wild type and T3SS-deficient Δ*escN* EHEC. Our findings confirmed previous observations that T3SS effectors inhibit NF-κB (Figure 6A, p<0.001, ANOVA; [20]). We discovered that deleting *nleH1*, but not *nleH2*, prevented EHEC from suppressing NF-κB activity (p = 0.002), indicating that NleH1 inhibits NF-κB-dependent transcription. By complementing the *nleH* mutants with corresponding NleH-FLAG plasmids, we also confirmed the dependence of alterations in RPS3/NF-κB-dependent luciferase activity upon NleH (Figure 6A).

We obtained similar data after transfecting 293T cells with NleH1 and NleH2 and stimulating these cells with TNF-α. By transfecting *nleH1*, we were able to inhibit NF-κB activity to ∼45% of native levels (Figure 6B; p<0.001, ANOVA). In contrast, transfecting *nleH2* stimulated NF-κB activity by ∼20%. We observed the differential activity of NleH1 vs. NleH2 even after we introduced additional RPS3 via transfection of RPS3-FLAG (Figure S6C). This activity was also dose-dependent with respect to the amount of transfected NleH (Figure S6D). By mutating the NleH1 K159 residue to alanine [NleH1(K159)], we eliminated NleH1-mediated NF-κB inhibition (Figure 6B).

Furthermore, neither NleH1 nor NleH2 altered CD25- or IL-2R-dependent luciferase expression. As these reporters are known to be RPS3-independent [18], these results further demonstrate the specificity of NleH for RPS3 (Figure 6C). Transfecting NleH2 increased the activity of an AP-1-dependent luciferase reporter, suggesting a potential target for this effector.

To extend our findings and to confirm that that activity of NleH1 is restricted to specific RPS3/NF-κB promoters, we performed RT-PCR to assess NleH regulation of several genes (IL-8, NFKBIA [42], and TNFIAP3 [43]) that are important to the innate response to infection and whose transcription is altered by rps3 knockdown [18]. IL-8 gene transcription was activated by infection with wild-type EHEC, as expected [20],[21]. This activation was further increased after infecting cells with either Δ*escN* or Δ*nleH1*, but not Δ*nleH2*, indicating that a T3SS effector [20],[21], most likely NleH1, inhibits IL-8 transcription (Figure 6D). Additionally, transcription of both the NFKBIA and TNFIAP3 genes were upregulated more in Δ*escN* and Δ*nleH1*, compared with wild-type and Δ*nleH2* infections, also suggesting a role for NleH1 in transcriptional inhibition of these RPS3-dependent genes.

To confirm further the role of NleH1 in inhibiting RPS3-dependent gene expression, we also performed transfection experiments. Transfecting rps3 siRNA into 293T cells inhibited IL-8 expression ∼4.5-fold (Figure 6E). Transfecting NleH1, but not NleH2 reproduced this phenotype. The transcription of both PLK1 and CENPE, two genes involved in NF-κB activation [44], was also inhibited by rps3 knockdown and by NleH1, but not by a sequence-scrambled siRNA or by NleH2. In contrast, transcription of IRF4, an RPS3-indepenent gene [18], was unaffected. Overall, these infection and transfection experiments suggest that NleH1 specifically inhibits RPS3-dependent gene expression.

We also analyzed site-directed NleH1 mutants to extend our findings that suggest NleH1 and NleH2 have differential influence on NF-κB activity. Similar to RPS3 nuclear abundance data obtained after transfection with different NleH1 point mutants (Figure 5E–F), co-transfecting NleH1(N40S,A42T,K45T) abolished NleH1 inhibitory activity, whereas mutating either the Y5 or the A100,A101,M102,I106 region did not prevent NleH1 from inhibiting NF-κB (Figure 6F).

We wished to determine the extent to which NleH1 and NleH2 are conserved among other A/E pathogens. We first noted that *C. rodentium* NleH was also able to reduce RPS3 nuclear abundance (Figure 5E–F) and to inhibit RPS3/NF-κB luciferase activity (Figure 6B). We cloned *nleH* from other O157:H7 and O88:H25 strains and tested their ability to inhibit RPS3/NF-κB luciferase activity by transfecting them into 293T cells (Figure S7). NleH cloned from other O157:H7 inhibited NF-κB activity to the same magnitude as EDL933 NleH1 (92–96% of EDL933 activity; data not shown). NleH cloned from O88:H25 strains also inhibited NF-κB activity, but to a lesser extent (74–78% of EDL933). Overall, these data demonstrate that NleH-mediated inhibition of RPS3/NF-κB transcriptional activity is not restricted to EDL933, but rather is conserved in non-O157 STEC and in *C. rodentium*.

### *E. coli* O157:H7 Δ*nleH1* is hypervirulent in gnotobiotic piglets

The 1-day-old gnotobiotic pig is a robust model organism for studying human EHEC disease [45]. Piglets challenged with EHEC develop diarrhea as a result of intimate bacterial attachment to the mucosal surfaces of the terminal ileum and large bowel. Infected piglets also develop fatal central nervous system (CNS) disease several days after inoculation [46], an important, yet underappreciated side effect of hemolytic uremic syndrome (HUS; [47]). Anatomically and physiologically similar to human children, piglets develop CNS symptoms and intestinal lesions develop over a similar time course following the onset of diarrhea [48]. EHEC infection of gnotobiotic piglets can be used to evaluate the contribution of virulence factors to host colonization and disease [9],[45].

**Figure 7:**
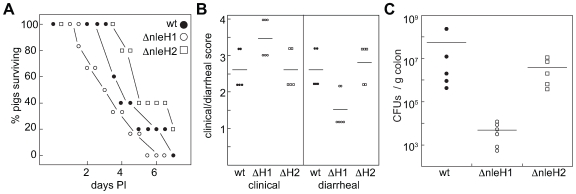
*E. coli* O157:H7 Δ*nleH1* is hypervirulent in gnotobiotic piglets. **A.** Survival analysis of gnotobiotic piglets as a function of time post-inoculation with EHEC strains possessing or lacking *nleH1* or *nleH2*. **B.** Quantification of piglet clinical outcome (left) and extent of diarrheal disease following EHEC challenge (right). **C.** Quantification of EHEC colonization of piglet colonic tissue (CFUs/g colon).

To measure the contribution of NleH1 and NleH2 to EHEC virulence *in vivo*, we orally infected gnotobiotic piglets at 24 h of age with 2*10^8^ CFUs of wild type EHEC EDL933, Δ*nleH1*, or Δ*nleH2* (n = 5−6/group). Piglets were observed every 4 h for signs of diarrhea, dehydration, and neurological signs of disease. Piglets infected with wild-type EHEC succumbed to infection over a ∼7-day period (Figure 7A, filled circles). Piglets infected with Δ*nleH1* (open circles), but not Δ*nleH2* (open squares), died more rapidly (Figure 7A; p = 0.004, Kruskal-Wallis test). Δ*nleH1* induced rapid and severe clinical signs of disease consistent with an inflammatory response (Figure 7B, left; p = 0.02), yet caused little diarrhea (Figure 7B, right; p = 0.01) and displayed significantly reduced colonization (Figure 7C; p = 0.001). In contrast, Δ*nleH2* had no effect on clinical outcome, though colonization of the piglet colon was reduced ∼10-fold.

## Discussion

PAMP recognition by TLRs transduces signals that activate and bind NF-κB to DNA (κB sites) within target gene promoters to regulate transcription by recruiting co-activators or co-repressors [18]. Activation typically proceeds via degradation of the inhibitory NF-κB chaperone IκBα, favoring the nuclear localization of NF-κB. Bacterial pathogens inject effector proteins into host cells to inhibit the innate immune response by interfering with NF-κB activation [49]. For example, *Shigella* OspG is a protein kinase that binds to ubiquitinylated ubiquitin-conjugating enzymes (E2s) to block degradation of IκBα, an event that ultimately inhibits NF-κB activity [26]. OspF is a phosphatase that targets MAPKs to prevent histone phosphorylation and dampen transcription at NF-κB-dependent promoters [50]. OspB and OspF also interact with the retinoblastoma protein, possibly to facilitate chromatin remodelling and diminish inflammatory cytokine production [51]. *Yersinia* YopJ inhibits the NF-κB pathway by inhibiting MAPKKs [52]. The *Salmonella* SseL effector is a deubiquitinating enzyme that impairs IκBα ubiquitination and degradation, thus reducing the production of NF-κB-dependent cytokines [53]. Mice lacking the p50 subunit of NF-κB (p50^−/−^) do not clear a *C. rodentium* infection within the duration of the experiment [54], indicating a critical role for NF-κB in host-defense against A/E pathogens. While the details have yet to be unravelled completely, EHEC T3SS effectors are suggested as key factors for bacterial inhibition of the host immune response [20].

Over a dozen ribosomal proteins that play significant roles in gene-specific transcriptional and translational regulation have been described (reviewed in [55],[56]). For example, RPL7 binds the Vitamin D receptor to regulate gene transcription [57]. Both RPL23 and RPS9 interact with the nucleolar chaperone protein B23. B23, also termed NPM/nucleophosmin/NO38, is a cofactor of the zinc-finger protein Miz1, a regulator of cell proliferation [55],[58]. RPL11 binds directly to Myc and inhibits its transcriptional activation activity [59]. Thus, the idea that ribosomal proteins also function in the nucleus to regulate transcription is well established, although until now, the targeting of these proteins by bacterial pathogens to subvert host cell functions was not.

By using several protein-interaction technologies, we determined that the translocated EHEC effectors NleH1 and NleH2 bind directly to the NF-κB non-Rel subunit, RPS3. NleH1 and NleH2 both interact with RPS3 through the binding of their N-termini to the N-terminal region of RPS3 that contains a putative nuclear localization signal (NLS). NleH1, but not NleH2, significantly reduced the nuclear abundance of RPS3, but had little influence upon other NF-κB subunits (e.g. p50 or p65). Using site-directed mutagenesis, we characterized a region of NleH1 (N40-K45) that is at least partially responsible for the inhibitory activity of this protein toward RPS3. By cloning and transfecting *nleH* from other O157 and non-O157 STEC, we demonstrated that NleH1 is present in other strains of significance to human health.

Although NleH1 and NleH2 are autophosphorylated proteins with Ser/Thr protein kinase activity, neither protein appeared to have the ability to phosphorylate RPS3. While the NleH1 K159 residue was non-essential for RPS3 binding, it was important to the ability of NleH1 to reduce RPS3 nuclear abundance, leaving open the possibility that other host proteins or translocated effectors may also be involved. Surprisingly, both the full-length and the isolated N-terminus of NleH1 (residues 1–139) inhibited RPS3 nuclear localization, while full-length NleH1 K159A site-directed mutant did not. Thus, NleH kinase activities may target another host protein with a role in RPS3 translocation, suggesting that NleH may be a multi-functional protein. We speculate that the K159A mutant may have an altered structure or have reduced ability to bind other cellular factors that affect RPS3 activities.

Others [24] and we have shown that NleH localizes to both host membranes and the cytosol following translocation. It is possible that the subcellular distribution of NleH may change to associate with the non-ribosomal pool of RPS3. It will be interesting to determine if mutating K159 somehow inhibits the ability of NleH to interact with a host protein needed for enrichment into the cytosol, a requirement that the isolated NleH N-terminus may not face.

The cellular factors governing the nuclear import of RPS3 are not completely understood. Its nuclear entry is thought to depend upon the phosphorylation of the T42 residue by the extracellular signal-regulated kinase (ERK) [38],[39]. However, other reports suggest that PKCδ phosphorylates RPS3 on S6 and T221, resulting in RPS3 nuclear mobilization to participate in DNA repair activities [40]. Furthermore, the role of RPS3 phosphorylation in its interaction with NF-κB has not yet been studied in detail [18]. As EPEC T3SS effectors have been shown to inhibit the phosphorylation-associated activation of ERK and p38 MAPK pathways [20], it will be of interest to learn if there may be a link between NleH and ERK/MAPK pathways, some of which might also alter RPS3 function [38],[39].

Despite the significant sequence identity among NleH1, NleH2 and OspG, a protein kinase which blocks degradation of IκBα [26] NleH1 or NleH2 did not appear to have the ability to alter IκBα degradation or to interact with the host ubiquitin machinery. However, it remains to be determined if NleH might associate with other E2 enzymes not studied in our assays. Notably, unlike NleH1 and NleH2, OspG does not possess the N-terminal region responsible for binding to RPS3, which might explain its differential activities and host targets.

Previous studies have elegantly shown that while A/E pathogens induce host IL-8 expression through both MAPK and TLR5-dependent activation of NF-κB, translocated effectors also down-regulate IL-8 at early stages of infection [20],[21]. However, only traditional NF-κB Rel proteins have been characterized in these studies. It is significant that these Rel proteins are associated with only ∼60% of canonical- or related-κB sites [60]. Thus, additional regulatory components, including RPS3, may contribute to high affinity DNA binding at various promoters [18]. Our results suggest that NleH1, but not NleH2, down-regulates NF-κB activity at RPS3-dependent, but not RPS-independent promoters. We propose that NleH1-reduction of RPS3 nuclear abundance in response to PAMP detection may explain previously documented findings that IL-8 expression is suppressed at early stages of infection, even in the context of NF-κB Rel protein nuclear translocation and increased DNA-binding activity [20],[21],[22],[23].

A *Shigella* OspG homolog in EHEC strain 11128 of *E. coli* serogroup O111 was recently described as a translocated effector [61]. This protein is somewhat distantly related (∼30% identity) to NleH1 or NleH2 and lacks the N-terminal domain of NleH that is responsible for binding to RPS3. In this work, EHEC OspG was not found to inhibit significantly NF-κB activity. These authors showed that an EHEC T3SS effector, but not OspG, inhibited p65 nuclear translocation and *E. coli* strain 13369 altered IκBα degradation at 1 h post-infection. It was also previously shown that NleH2, rather than NleH1, is encoded by EHEC strains of the O111 serotype [62]. As we have shown that NleH1, but not NleH2, inhibits the contribution of RPS3 to NF-κB activity, it is perhaps not surprising that inhibition of NF-κB was not observed in the O111 strains recently examined [61]. Consistent with these studies [61], we also failed to observe a role for NleH in inhibiting p65 nuclear translocation. However, our analysis of NleH is limited to RPS3-dependent κB promoters and we consider it likely that other T3SS effectors will be discovered to inhibit NF-κB through other mechanisms.

While we did not observe NleH2 to inhibit RPS3 function, it may influence NF-κB through an alternative mechanism or affect other host signal transduction networks. Interestingly, NleH2 increased the activity of an AP-1-dependent luciferase reporter. EHEC infection has been shown to induce the phosphorylation of multiple MAPKs, including ERK1/2, p38, and JNK, which in turn induce AP-1 activation at later stages of infection [63]. There are multiple transcription factor binding sites in the IL-8 promoter, including both AP-1 and NF-κB [64]. NleH1 and NleH2 may potentially target different host signal transduction pathways to contribute to the suppression of host innate responses to infection.

It was previously reported that an ‘espG/orf3Δcore’ EPEC mutant deficient in ‘EspG/Orf3/EspH/CesF/Map/CesT/Tir/Intimin’ did not differ from wild-type EPEC in its ability to inhibit IL-8 secretion from Caco-2 cells [20]. Because this mutant also lacked the chaperone CesT, it was predicted to be deficient in translocation of other effectors, including NleH [20],[65]. Thus, it was postulated that inhibition of IL-8 secretion might be due to a different non-LEE T3SS-dependent effector [20]. However, others reported that deletion of *nleH* in *C. rodentium* reduced TNF-α expression in infected mice, as compared with wild-type *C. rodentium* infection [25]. These contrasting results may also be a function of the different assay systems, cell types, and animal models employed. For example, it has been reported that the in vivo effect of NleH may be variable with respect to the host animal under investigation [25]. Deleting both *nleH1* and *nleH2* from *E. coli* O157:H7 caused increased shedding compared with the parental strain in Friesian bull calves, yet had a reduced competitive advantage in mixed infections of lambs [25].

Our analysis of EHEC strains deleted for *nleH1* and *nleH2* in gnotobiotic piglets support a role for NleH1 in modulating the host innate response to infection. Previous studies have reported that severe HUS correlates with increased inflammation induced by STEC infection [66]. Chemokines produced in response to bacterial PAMPs have been reported to lead to the stimulation and subsequent basolateral-to-apical transmigration of neutrophils, inducing concomitant translocation of Shiga-like toxins in the opposite direction [67]. Shiga toxin attachment to monocytes and macrophages can stimulate TNF-α, IL-1 and IL-6 secretion, subsequently increasing globotriaosylceramide (Gb3) expression on endothelial cell membranes [68]. Intimin-negative STEC that lack the LEE (and thus are unlikely to translocate Nles) induce higher neutrophil migration and higher IL-8 secretion compared with intimin-positive STEC [67]. It should be noted that the primary cause of death in the gnotobiotic piglet model we employed is a manifestation of systemic intoxication, rather than diarrheal disease. As deleting *nleH1* relieved the inhibition of NF-κB-dependent IL-8 gene expression in our in vitro assays, it is possible that deleting *nleH1* could lead to greater neutrophil activation and Stx influx into the circulatory system.

Our findings suggest a novel mechanism for bacterial manipulation of the host response and demonstrate the direct binding of a bacterial effector to an NF-κB subunit. NF-κB is a major regulator of not only acute antimicrobial responses, but also adaptive immune responses. A growing number of cellular factors that govern the selective binding of NF-κB to different promoters [18],[69],[70],[71], combined with the discovery of novel bacterial effectors [6],[7], provide us with significant opportunity to understand how pathogens subvert the host response to infection.

## Materials and Methods

### Ethics statement

All animal experiments were performed according to Institutional Animal Care and Use Committee-approved protocols (Animal Welfare Assurance no. A3958-01).

### Materials

The TEM-1 fusion cloning vector pCX340 was a generous gift from E. Oswald. Chemicals were used according to manufacturers' recommendations and were obtained from Sigma, except for the following: myelin basic protein (New England Biolabs), Erk1 kinase (Cell Signaling), QuikChange Site-Directed Mutagenesis Kit (Stratagene), Dual-Luciferase Reporter Assay System (Promega), CCF2-AM Loading Kit (Invitrogen), Ni-NTA agarose (Qiagen), protein G agarose (Fisher), TNF-α (Genscript), RPS3 siRNA (Santa Cruz Biotechnology), and FuGene (Roche). Antibodies were obtained from the following sources: calnexin, His, FLAG, HA, β-actin, and tubulin (Sigma), phospho-Ser/Thr (Abcam), RPS3 (PrimmBiotech Inc.), RPS16 (Abnova), TEM (QED bioscience), p50/p105, p65, IκBα ubiquitin (Genscript), PARP (BD Biosciences), and phospho-IκBα (Cell Signaling).

### Bacterial strains, plasmids, and oligonucleotides

The bacterial strains and plasmids used in this study are described in Table 1. The oligonucleotides used in this study are described in Table 2. The deletion of individual *nleH1* and *nleH2* genes, and the construction of a Δ*nleH1*Δ*nleH2* mutant was performed using lambda Red-mediated mutagenesis [41] in *E. coli* O157:H7 EDL933 by replacing *nleH1* and/or *nleH2* with a kanamycin resistance marker, using plasmid pKD46 to generate the deletion cassettes. Gene replacements were confirmed with multiple PCRs and by RT-PCR. The bacterial growth kinetics, bacterial adherence to host cells, and Tir translocation into host cells did not differ significantly among *nleH* mutants and the parental strains.

**Table 1:** Strains and plasmids utilized in this study.

Strain or plasmid	Description	Reference
*Strains*		
*Escherichia coli* BL21(DE3)	*E. coli* F^-^ *ompT hsdSB* (r_B_^-^m_B_^-^) *gal dcm* (DE3)	Novagen
BL21(DE3)/pNleH1-pET28a	His-NleH1	This study
BL21(DE3)/pNleH2-pET28a	His-NleH2	This study
BL21(DE3)/pNleH1(K159A)-pET28a	His-NleH1 K159A mutant	This study
BL21(DE3)/pNleH2(K169A)-pET28a	His-NleH2 K169A mutant	This study
EPEC E2348/69	wild type *E. coli* O127:H6 isolate	[73]
E2348/69Δ*escN*	EPEC T3SS mutant	[74]
E2348/69/pNleH1-FLAG	EPEC expressing NleH1-FLAG	This study
E2348/69 Δ*escN*/pNleH1-FLAG	T3SS EPEC expressing NleH1-FLAG	This study
E2348/69/pNleH2-FLAG	EPEC expressing NleH2-FLAG	This study
E2348/69 Δ*escN*/pNleH2-FLAG	T3SS EPEC expressing NleH2-FLAG	This study
E2348/69/pNleH1-TEM	EPEC expressing NleH1-TEM	This study
E2348/69 Δ*escN*/pNleH1-TEM	T3SS EPEC expressing NleH1-TEM	This study
E2348/69/pNleH2-TEM	EPEC expressing NleH2-TEM	This study
E2348/69 Δ*escN*/pNleH2-TEM	T3SS EPEC expressing NleH2-TEM	This study
E2348/69/pNleH1(K159A)-TEM	EPEC expressing NleH1(K159A)-TEM	This study
E2348/69 Δ*escN*/pNleH1(K159A)-TEM	T3SS EPEC expressing NleH1(K159A)-TEM	This study
E2348/69/pNleH2(K169A)-TEM	EPEC expressing NleH2(K169A)-TEM	This study
E2348/69 Δ*escN*/pNleH2(K169A)-TEM	T3SS EPEC expressing NleH2(K169A)-TEM	This study
E2348/69/pTEM	EPEC expressing TEM	This study
E2348/69 Δ*escN*/pTEM	T3SS EPEC expressing TEM	This study
EPEC UMD207	EPEC *bfp-eae-*	[75]
EPEC UMD207/pNleH1-FLAG	UMD207 expressing NleH1-FLAG	This study
EPEC UMD207/pNleH2-FLAG	UMD207 expressing NleH2-FLAG	This study
EPEC UMD207/pNleH1(K159A)-FLAG	UMD207 expressing NleH1(K159A)-FLAG	This study
EPEC UMD207/pNleH2(K169A)-FLAG	UMD207 expressing NleH2(K169A)-FLAG	This study
EPEC UMD207/pFLAG	UMD207 expressing FLAG	This study
EPEC UMD207/pnleF-FLAG	UMD207 expressing NleF-FLAG	[9]
EHEC EDL933	wild type *E. coli* O157:H7 isolate	CDC
EDL933 Δ*escN*	EHEC T3SS mutant	J. Puente
EDL933 Δ*nleH1*	EHEC *nleH1* deletion	This study
EDL933 Δ*nleH2*	EHEC *nleH2* deletion	This study
EDL933 Δ*nleH1*Δ*nleH2*	EHEC *nleH1nleH2* deletion	This study
EDL933/pNleH1-FLAG	EHEC expressing NleH1-FLAG	This study
EDL933/pNleH2-FLAG	EHEC expressing NleH2-FLAG	This study
EDL933/pFLAG	EHEC expressing FLAG	This study
EDL933 Δ*nleH1*/pNleH1-FLAG	EHEC *nleH1* deletion expressing NleH1-FLAG	This study
EDL933 Δ*nleH2*/pNleH2-FLAG	EHEC *nleH2* deletion expressing NleH2-FLAG	This study
EDL933 Δ*nleH1*/pNleH1-FLAG(K159A)	EHEC *nleH1* deletion expressing NleH1(K159A)-FLAG	This study
EDL933 Δ*nleH2*/pNleH2-FLAG(K169A)	EHEC *nleH2* deletion expressing NleH2(K169A)-FLAG	This study
*Plasmids*		
pCX340	TEM-1 reporter plasmid	[30]
NleH1-TEM	NleH1-TEM	This study
NleH2-TEM	NleH2-TEM	This study
pET28a	Bacterial hexahistidine fusion expression	Novagen
NleH1-pET28a	His-NleH1	This study
NleH2-pET28a	His-NleH2	This study
NleH1(K159A)-pET28a	His-NleH1 K159A mutant	This study
NleH2(K169A)-pET28a	His-NleH2 K169A mutant	This study
pFLAG-CTC	Bacterial FLAG fusion protein expression	Sigma
NleH1-pFLAG-CTC	NleH1-FLAG	This study
NleH2-pFLAG-CTC	NleH2-FLAG	This study
NleH1(K159A)-pFLAG-CTC	NleH1(K159A)-FLAG	This study
NleH2 (K169A)-pFLAG-CTC	NleH2(K169A)-FLAG	This study
NleF-pFLAG-CTC	NleF-FLAG	[9]
p3XFLAG	Mammalian FLAG fusion expression	Sigma
RPS3-p3XFLAG	FLAG-RPS3	[18]
RPS3(1-101)-p3XFLAG	FLAG RPS3 (AAs 1–101)	[18]
RPS3(ΔKH)-p3XFLAG	FLAG RPS3 (lacking KH domain AAs 41–111)	[18]
RPS3(41-243)-p3XFLAG	FLAG RPS3 (AAs 41–243)	[18]
RPS3(112-243)-p3XFLAG	FLAG RPS3 (AAs 112–243)	[18]
κB (5X)-luc	Firefly luciferase driven by RPS3/NF-κB-dependent κB site	Promega
pTKRL-luc	Renilla luciferase	Promega
IL-2R-luc	Firefly luciferase driven by IL-2R promoter	[18]
CD25-luc	Firefly luciferase driven by CD25-promoter	[18]
AP-1-luc	Firefly luciferase driven by AP-1-promoter	[18]
pKD46	Lambda Red mediated mutagenesis	[41]
VN	Venus fluorescence protein (AAs 1–173)	[76]
VC	Venus fluorescence protein (AAs 155–238)	[76]
VN-actin	Venus 1–173 fused to human actin	[76]
VC-actin	Venus 155–238 fused to human actin	[76]
VN-NleH1	Venus 1–173 fused to NleH1	This study
VC-NleH1	Venus 155–238 fused to NleH1	This study
VN-NleH2	Venus 1–173 fused to NleH2	This study
VC-NleH2	Venus 155–238 fused to NleH2	This study
VN-RPS3	Venus 1–173 fused to RPS3	This study
VC-RPS3	Venus 155–238 fused to RPS3	This study
VN-NleH1(1–129)	Venus 1–173 fused to NleH1 AAs 1–129AA	This study
VN-NleH1(130–293)	Venus 1–173 fused to NleH1 AAs 130–293 AA	This study
VN-NleH2(1–139)	Venus 1–173 fused to NleH2 AAs 1–139AA	This study
VN-NleH2(140–303)	Venus 1–173 fused to NleH2 AAs 140–303	This study
VN-Citro-NleH	Venus 1–173 fused to *C. rodentium* NleH	This study
VN-NleH1(Y5S)	Venus 1–173 fused to NleH1(Y5S)	This study
VN-NleH1(N40S,A42T,K45T)	Venus 1–173 fused to NleH1(N40S,A42T,K45T)	This study
VN-NleH1 (A100T,A101T,M102I,I106S)	Venus 1–173 fused to NleH1(A100T,A101T,M102I,I106S)	This study

**Table 2:** Oligonucleotides used in this study.

Primer	Sequence (5′-3′)
NleH1 XhoI-f -FLAG/HA	G_2_CTCGAGATGT_2_ATCGC_2_ATAT_2_CTGTA_3_T_3_G_3_
NleH1 BamHI-f-His	G_4_ATC_2_ATGT_2_ATCGC_2_ATAT_2_CTGTA_3_T_3_G_3_
NleH1 KpnI-r-FLAG	C_2_G_2_TAC_2_A_2_T_4_ACT_2_A_2_TAC_2_ACACTA_2_TA_2_GATCT_2_GCT_3_C_2_GC_2_
NleH1 XhoI-r-His	C_3_TCGAGA_2_T_4_ACT_2_A_2_TAC_2_ACACTA_2_TA_2_GATCT_2_GCT_3_C_2_GC_2_
NleH2 XhoI-f-FLAG/HA	G_2_CTCGAGATGT_2_ATCGC_3_TCT_2_CTATA_3_T_3_G_3_
NleH2 BamHI-f-His	G_4_ATC_2_ATGT_2_ATCGC_3_TCT_2_CTATA_3_T_3_G_3_
NleH2 KpnI-r-FLAG	C_2_G_2_TAC_2_TATCT_2_ACT_2_A_2_TACTACACTA_2_TA_2_GATC_2_AGCT_3_C_2_
NleH2 XhoI-r-His	C_3_TCGAGTATCT_2_ACT_2_A_2_TACTACACTA_2_TA_2_GATC_2_AGCT_3_C_2_
NleH2 XmaI-r	C_5_G_3_TATCT_2_ACT_2_A_2_TACTACACTA_2_TA_2_GATC_2_AGCT_3_C_2_
NleH1 XhoI-f	CTCGAGATGT_2_ATCGC_2_ATAT_2_CTG
NleH1 NdeI-f-TEM	GCCATATGT_2_ATCGC_2_ATAT_2_CTGTA_3_T_3_G_3_
NleH1 KpnI-r-TEM	GCG_2_TAC_2_A_2_T_4_ACT_2_A_2_TAC_2_ACACTA_2_TA_2_G
NleH2 NdeI-f-TEM	GC_2_ATATGT_2_ATCGC_3_TCT_2_CTATA_3_T_3_G_2_
NleH2 KpnI-r-TEM	GCG_2_TAC_2_TATCT_2_ACT_2_A_2_TACTACACTA_2_TA_2_G
NleH1 K159A-f	GCA_2_CA_4_GTC_2_TG_2_CGATGT_3_ACTACATCTC
NleH1 K159A-r	GAGATGTAGTA_3_CATCGC_2_AG_2_ACT_4_GT_2_GC
NleH2 K169A–f	CA_2_CA_4_GTGT_2_G_2_CGATGT_3_ACTATATCTC
NleH2 K169A–r	GAGATATAGTA_3_CATCGC_2_A_2_CACT_4_GT_2_G
RPS3 XhoI-f-HA	GC_2_TCGAGATG_2_CAGTGCA_3_TATC_2_A_2_GA_2_GAG_2_
RPS3 NotI-r-HA	GCGCG_2_C_2_GCT_2_ATGCTGTG_4_ACTG_2_CTG_4_C
NleH1 NotI-r-HA	C_2_GCG_2_C_2_GCT_2_A_3_T_4_ACT_2_A_2_TAC_2_ACACTA_2_TA_2_G
NleH2 NotI-r-HA	C_2_GCG_2_C_2_GCT_2_ATATCT_2_ACT_2_A_2_TACTACAC
NleH1 pKD4-f	TGTATGT_2_ATCGC_2_ATAT_2_CTGTA_3_T_3_G_3_ATGT_2_CTGTGTAG_2_CTG_2_AGCTGCT_2_CG
NleH1 pKD4-r	GATA_4_T_2_ACTA_3_T_4_ACT_2_A_2_TAC_2_ACACTA_2_TA_2_GCATATGA_2_TATC_2_TC_2_T_2_AG
NleH2 pKD4-f	C_2_TCT_2_CTATA_3_T_3_G_3_ATGT_2_CATG_2_A_2_T_2_CT_3_A_2_CGTGTAG_2_CTG_2_AGCTGCT_2_C
NleH2 pKD4-r	A_2_TACTACACTA_2_TA_2_GATC_2_AGCT_3_C_2_TC_2_GTGATA_2_GCATATGA_2_TATC_2_TC_2_T_2_A
NleH1 N-term-NotI-r-HA	GCGCG_2_C_2_GCT_2_A_2_TAGTC_2_ACACTCG_2_TA_2_CTCTG_2_CG
NleH1 C-term-XhoI-f-HA	GC_2_TCGAGATGA_2_CAG_2_T_2_GTCAGTAC_2_TG_2_TA_2_TG
NleH2 N-term-NotI-r-HA	GCGCG_2_C_2_GCT_2_AGT_2_GCTA_2_C_3_TCG_2_CA_2_CTCTGAT_2_G
NleH2 C-term-XhoI-f-HA	GC_2_TCGAGATGA_2_TA_3_TCAC_3_GTGC_2_AG_2_TA_2_TG
NleH1 Y5S-f	ATGT_2_ATCGC_2_ATCT_2_CTGTA_3_T_3_G_3_ATGT_2_C
NleH1 Y5S-r	GA_2_CATC_3_A_3_T_3_ACAGA_2_GATG_2_CGATA_2_CAT
NleH1 N40S-f	GC_2_GT_2_CAT_2_CTGATAGCG_3_ACGCA_2_GTA_2_CG_2_T_2_G_2_C
NleH1 N40S-r	GC_2_A_2_C_2_GT_2_ACT_2_GCGTC_3_GCTATCAGA_2_TGA_2_CG_2_C
NleH1 A100T-f	CAC_2_AGTGAGCACGACTATAGA_3_GAG_2_CTCTGT_2_CAT_2_CGA_2_C
NleH1 A100T-f	GT_2_CGA_2_TGA_2_CAGAGC_2_TCT_3_CTATAGTCGTGCTCACTG_2_TG
GAPDH RT-f	AC_2_AG_2_TG_2_TCTC_2_TCTGACT_2_C
GAPDH RT-r	GTG_2_TCGT_2_GAG_2_GCA_2_TG
IL-8 RT-f	CTG_2_C_2_GTG_2_CTCTCT_2_G
IL-8 RT-r	C_2_T_2_G_2_CA_4_CTGCAC_2_T_2_
NFKBIA RT-f	C_5_TACAC_2_T_2_GC_2_TGTG
NFKBIA RT-r	TCAGCAC_3_A_2_G_2_ACAC_2_A_2_
TNFIAP3 RT-f	T_2_GC_2_TCATGCATGC_2_ACT_2_
TNFIAP3 RT-r	AGCA_3_GC_4_GT_3_CA_2_CA
PLK1 RT-f	GAG_2_AG_2_A_3_GC_3_TGACTGA
PLK1 RT-r	GCAGC_2_A_2_GCACA_2_T_3_GC
CENPE RT-f	GC_2_A_2_GACG_2_AC_2_A_2_GCA
CENPE RT-r	TGCT_2_GTAGCTGCGCT_2_C_2_T
IRF4 RT-f	TG_2_T_2_GC_2_AG_2_TGACAG_2_A_2_
IRF4 RT-r	TC_2_AG_2_T_2_GCTG_2_CGTCATA

### Effector translocation assays

NleH-TEM fusions were constructed by cloning *nleH1 and nleH2* into pCX340. For assaying NleH-TEM translocation, plasmids were introduced into wild type and Δ*escN* EPEC and used to infect HeLa cells for 4 h in DMEM supplemented with 1 mM IPTG. Cells were washed and loaded with CCF2/AM. Fluorescence was evaluated after cells were illuminated at 409 nm [30]. NleH-FLAG fusions were constructed by cloning *nleH1* and *nleH2* into pFLAG-CTC (Sigma). HeLa cell monolayers were infected with EPEC strains (MOI ∼100) for 4 h, washed, trypsinized, and resuspended in homogenization buffer (3 mM imidazole [pH 7.4], 250 mM sucrose, 0.5 mM EDTA). Cells were mechanically disrupted by vigorous passage through 22- and 27-gauge needles. Low-speed centrifugation (3,000 g, 15′) was applied to the homogenate to pellet the bacteria, unbroken HeLa cells, host nuclei, and cytoskeletal components. The supernatant was then subjected to ultracentrifugation (100,000 g, 1 h) to separate insoluble host cell membranes from soluble cytosolic proteins [24]. The resulting fractions were resolved by SDS-PAGE (10% polyacrylamide) and transferred to nitrocellulose for Western blot analysis.

### Protein purification

*nleH1* and *nleH2* were cloned into pET-28a and expressed in *E. coli* BL21(DE3). 250 ml of bacterial culture were grown to an OD_600_ of 0.6, when IPTG was added to 1 mM. After 4 h additional growth, cells were pelleted and resuspended in 25 ml cold PBS. Cells were sonicated and centrifuged to clarify the supernatant. The supernatant was added to pre-equilibrated Ni-NTA agarose and incubated for 2 h. The slurry was washed 5 times with 60 mM imidazole, 500 mM NaCl, 20 mM Tris HCl pH 7.9, eluted in 1 M imidazole, 1 M NaCl, 40 mM Tris HCl pH 7.9, and analyzed on 12% SDS-PAGE.

### Affinity columns

Thirty confluent 150 mM dishes of HeLa cells were washed with PBS and lysed in 20 ml of binding buffer [150 mM NaCl, 20 mM Tris (pH 8.0), 1% Triton X-100]. After centrifugation (5,000 g, 4°C, 10′), 10 ml of the supernatant was transferred to a 15 ml conical tube containing 300 µl Ni-NTA agarose in the presence or absence of 500 µg purified His-NleH1. After incubation for 1 h at 4°C, the columns were washed with binding buffer and then eluted with increasing concentrations of imidazole (0–300 mM). Fractions were analyzed by SDS-PAGE and staining with Coomassie Blue G-250.

### Protein identification by LC-ESI-MS/MS

Bands excised from protein gels were digested in-gel with trypsin at 37°C overnight. The tryptic peptide solution was transferred to a microcentrifuge tube, extracted with 1% formic acid, 2% acetonitrile in water, followed by extraction with 50% acetonitrile. Both extracts were combined, concentrated, and suspended in 3% acetonitrile, 0.1% formic acid. Peptide analysis was performed using LC-ESIMS/MS. Peptides were desalted in-line and concentrated with RP-Trap Symmetry300 C18 column, 5 µm NanoEase (Waters), and separated using a C18 RP PepMap capillary column on a CapLC (Dionex). The eluted ions were analyzed by one full precursor MS scan (400–1500 m/z), followed by four MS/MS scans of the most abundant ions detected in the MS scan. Spectra were obtained in the positive ion mode with a nano ESI-Q-Tof micro mass spectrometer (Micromass). A peak list (PKL format) was generated to identify +1 or multiple charged precursor ions from the mass spectrometry data file. Mascot server v2.2 (www.matrix-science.com) in MS/MS ion search mode was applied to conduct peptide matches (peptide masses and sequence tags) and protein searches against NCBInr v20080110.

### Immunoprecipitation and immunoblotting

Bacterial strains were grown in Luria–Bertani (LB) broth, at 37°C in 5% CO_2_ without shaking. HeLa cells were grown in Dulbecco's minimal Eagle medium (DMEM) supplemented with 10% heat-inactivated fetal calf serum (FCS). Cell culture media was replaced with DMEM 3 h prior to infection. Overnight bacterial cultures were diluted 1∶10 into DMEM, incubated 3 h at 37°C in 5% CO_2_ (to ‘pre-activate’ the T3SS; [72]) and used to infect two 150 mm dishes of ∼80% confluent HeLa cells at a multiplicity of infection of ∼50. Cells were harvested after a 4 h infection by washing them 3 times with PBS to remove bacteria. Cells were scraped into PBS, pooled, centrifuged (1,500 g, 5′), resuspended in PBS, and recentrifuged. The supernatant was removed and cells were lysed in RIPA [150 mM NaCl, 50 mM Tris pH 8.0, 0.4 mM EDTA, 10% glycerol, 1% Nonident P-40 (NP-40)], followed by brief vortexing and rotation for 30′ at 4°C. The supernatant was transferred to a tube containing protein G sepharose (prewashed in RIPA) and incubated for 1 h at 4°C. Samples were centrifuged (1,500 g, 5′), transferred to prewashed α-FLAG resin, and incubated with rotation overnight at 4°C. FLAG resin was washed with RIPA and resuspended in 500 µl RIPA (without NP-40). The resin was centrifuged (2,000 g, 5′) and the supernatant was removed with a 29-gauge needle. NleH-FLAG and associated proteins were eluted from the resin by adding 72 µg FLAG peptide (in 200 µl RIPA) and incubating with rotation for 1 h at 4°C. The resin was centrifuged (2,000 g, 5′), and the supernatant was transferred to a fresh tube, precipitated with trichloroacetic acid (TCA), and resuspended in SDS-PAGE buffer. IP samples were interrogated for the presence of NleH-FLAG and RPS3 by Western blotting.

Equal amounts of protein from cell lysates (50 µg) and IPs (5 µg) were separated by SDS-PAGE, transferred to nitrocellulose, blocked in Odyssey blocking buffer, double-probed overnight with mouse-α-FLAG and rabbit-α-RPS3 1° antibodies, washed in PBS, and then incubated for 30′ with Alexa Fluor 680/750 goat-α-rabbit and goat-α-mouse 2° antibodies at room temperature. After rinsing in PBS, blots were imaged with an Odyssey infrared imaging system (Li-Cor). For immunoprecipitation experiments with transfected RPS3 and NleH plasmids, 293T cells were incubated in 6-well plates and transfected with 1 µg total plasmid using FuGene.

### Bimolecular fluorescence complementation

HeLa cells were cultured in DMEM supplemented with 10% FCS and co-transfected with a pair of BiFC plasmids (100–250 ng each) representing NleH and RPS3 truncations cloned as fusions to the N- or C-terminus of Venus eYFP (designated VN and VC). The fluorescence intensities derived from BiFC (due to effector-host protein binding) were measured after 48 h incubation using appropriate filters (excitation: 500/20 nm; emission: 535/30 nm).

### Immunofluorescence microscopy

HeLa and 293T cells were grown on glass coverslips in 24-well tissue culture plates and infected for 4 h with overnight cultures of indicated bacterial strains (MOI∼20–50). After infection, cells were washed 3 times in PBS containing Ca^2+^/Mg^2+^ and fixed in 2.5% paraformaldehyde. Cells were permeabilized in 0.1% saponin in PBS, blocked with 5% goat serum, and incubated with α-FLAG (1∶1,000), and α-RPS3 (1∶200) primary antibodies for 1 h at room temperature. The cells were washed with PBS and probed with Alexa Fluor 488- and 594-conjugated secondary antibodies (1∶1,000, 1 h) and DAPI (1 µg/ml, 2′). For experiments involving transfection, 1 µg total plasmid suspended in Fugene was used and cells were typically immunostained 48 h post-transfection. Coverslips were mounted in Mowiol and samples were visualized using a LSM 510 Laser Scanning Microscope (Carl Zeiss).

### In vitro kinase assays

Kinase assays were performed by incubating His-NleH proteins (10 ng/µl) with myelin basic protein (MBP, 100 ng/µl) in 50 mM Tris-HCl, pH 7.6, 5 mM MgCl_2_, 1 mM DTT, 1 mM ATP, at 30°C for 1 h. Phosphorylation was monitored by separating the samples by SDS-PAGE and Western blotting with an a-phospho-Ser/Thr-specific antibody [40] and Pro-Q staining. As a positive control in the assay, ERK1 was used at a concentration of 5 ng/µl, as it is known to phosphorylate MBP [38].

### RPS3 nuclear translocation

Cytosolic and nuclear protein extracts were prepared from HeLa or 293T cells transfected with NleH plasmids or infected with *nleH* mutants as previously described [18]. TNF-α stimulation (1 h, 100 ng/ml) was used to promote RPS3 translocation into the nucleus. Data were analyzed by Western blotting for nuclear RPS3. PARP and tubulin were used to normalize the protein concentrations of nuclear and cytoplasmic fractions, respectively.

### Luciferase assays

HeLa or 293T cells were co-transfected at a ratio of 10∶1 (1.0 µg total DNA) with a firefly luciferase construct driven by a consensus κB site, together with the renilla luciferase pTKRL plasmid (Promega), cultured for 36–48 h, and then infected with EHEC strains for 3 h, in the presence or absence of TNF-α stimulation (κB, CD25, IL-2R reporters) or PMA (AP-1 reporter) or silencing with rps3 siRNA. Cells were lysed with passive lysis buffer and lysates were analyzed by using the Dual-Luciferase Kit (Promega) with firefly fluorescence units (FU) normalized to renilla FU. The fold-induction was calculated as [relative FU stimulated)/(relative FU unstimulated] samples. Luciferase assays were performed in triplicate with at least three independently transfected cell populations.

### RT-PCR

cDNA was prepared from 1 µg RNA by using the Superscript First Strand System (Invitrogen) with oligo (dT) primer. Real-time PCR was performed in triplicate using a SYBR Green PCR Master Mix (Ambion) in a Fast 7500 sequence-detection system (Applied Biosystems). Relative transcription levels were calculated by using the ΔΔCt method.

### Gnotobiotic piglet infections

Gnotobiotic piglets were delivered into germ-free incubators through sterile closed hysterotomy of pregnant sows. Piglets were separated into individual compartments without regard to sex, fed individually with a sterile commercial piglet formula (SPF-Lac; PetAg, Inc.), and inoculated at 24 h of age with 3 ml tryptic soy broth containing 1*10^8^ CFUs of wild type, D*nleH1*, or D*nleH2 E. coli* O157:H7 EDL933. Piglets were observed every 4 h for signs of diarrhea, dehydration, and neurological signs of disease (head tilt, circling, lethargy, inability to stand, lateral recumbency and paddling). When piglets developed severe dehydration, lethargy or CNS disease, they were euthanized and subjected to postmortem examination. Animals that did not become lethargic or dehydrated were euthanized after 8 d. Spiral colon specimens were collected at necropsy, split longitudinally, rinsed in PBS to remove feces, diluted 1∶10 (*w/v*) in PBS, ground, normalized to tissue weight, serially diluted, and cultured to quantify the extent of intestinal colonization. Data were normalized by tissue weight.

### Statistical analyses

NleH translocation, BiFC, RPS3 immunoblotting, luciferase, and RT-PCR assays were analyzed statistically using one-way ANOVA. RPS3 immunofluorescence data were analyzed with t-tests. Gnotobiotic piglet data were analyzed with the Kruskal-Wallis Test. p-values<0.05 were considered significant.

## Supporting Information

Figure S1 NleH amino acid sequences and T3SS-dependent translocation. A. *E. coli* EDL933 NleH1 (Z0989) and NleH2 (Z6021) amino acid sequences. Asterisks indicate identical residues. Amino acids differing between NleH1 and NleH2 are indicated in red. The lysine residue implicated in autophosphorylation activity is depicted in blue. B. Immunoblotting of bacterial lysates for NleH1- and NleH2-TEM expression in wild type (wt) or T3SS-deficient Δ*escN* EPEC (T3SS). Blots were probed with α-TEM antibody. C. β-lactamase activity (arbitrary units) in HeLa cells loaded with CCF2/AM substrate and infected for 4 h with wt or Δ*escN* EPEC (T3SS) strains expressing NleH1- or NleH2-TEM fusions. Asterisks indicate significantly different β-lactamase activity compared with uninfected samples (p<0.05, ANOVA). D. Immunoblot analysis of cytoplasmic and membrane HeLa cell fractions following infection with EPEC strains expressing NleH1- or NleH2-FLAG. Blots were probed with α-FLAG, α-tubulin, and α-calnexin antibodies.
(0.46 MB PDF)

Figure S2 Binding specificity of NleH. A. Affinity enrichment of HeLa proteins with affinity for His-NleH1. HeLa cell lysates were incubated with purified His-NleH1 pre-bound to Ni-NTA agarose, eluted with imidazole, and analyzed by SDS-PAGE. Samples included HeLa lysate + His-NleH1 (lane 1), His-NleH1 (lane 2), and HeLa lysate (lane 3). Bands identified by mass spectrometry as NleH1 and RPS3 are indicated. B. NleH does not bind RPS16. HeLa cells were infected with EPEC expressing NleH1-FLAG and immunoprecipitated with α-RPS3 (left) or α-RPS16 (right) antibodies. The top and middle panels depict the abundance of RPS3 and RPS16 in the cell lysate, whereas the bottom panel depicts an α-FLAG immunoblot of the immunoprecipitated samples. Similar results were obtained with NleH2-FLAG. C. NleF does not bind RPS3. HeLa cells were infected with EPEC expressing FLAG (left) or NleF-FLAG (right) and immunoprecipitated with α-RPS3 antibody. The top panel depicts RPS3 in the cell lysates whereas the middle and bottom panels depict samples immunoprecipitated with α-FLAG antibody and subsequently immunoblotted for FLAG and RPS3, respectively.
(1.56 MB PDF)

Figure S3 NleH1 and NleH2 are autophosphorylated Ser/Thr protein kinases. A. Autophosphorylation assay of His-NleH1 and NleH2, and site-directed mutants NleH1(K159A) and NleH2(K169A). Blots were stained with Pro-Q. B. Phosphorylation of myelin basic protein (MBP) by wild-type NleH1 and NleH2, but not the site-directed mutants NleH1(K159A) and NleH2(K169A). Blots were probed with α-His and α-phospho-Ser/Thr antibodies.
(0.38 MB PDF)

Figure S4 T3SS effector(s) inhibit RPS3 nuclear translocation. A. Immunofluorescence microscopy analysis of RPS3 nuclear abundance in HeLa cells infected with wild type (wt) or Δ*escN* EHEC. B. Quantification of the % of cells containing predominantly nuclear RPS3 (n = 100 cells). Asterisks indicate significantly different compared with wild-type infection (p<0.05, t-test).
(0.95 MB PDF)

Figure S5 NleH1 reduces the nuclear abundance of RPS3. A. Quantification (n = 4) of the fold-increase in nuclear p65 as assessed from immunoblotting (depicted in Figure 5A), in the absence (open bars) or presence (black bars) of TNF-α stimulation. p65 signal intensity was normalized to PARP. B. Immunofluorescence microscopy analysis of NleH and RPS3 localization as a function of TNF-α stimulation. HeLa cells were infected for 3 h with EPEC strains expressing NleH1- or NleH2-FLAG, treated with TNF-α (100 ng/ml) for 1 h, and stained with DAPI (blue), a-FLAG (green), and a-RPS3 (red) monoclonal antibodies. C. Immunoprecipitation of nuclear extracts with α-p65 antibody. Immunoprecipitated samples were immunoblotted for p65 and RPS3 in samples transfected with the indicated plasmids, in the absence or presence of TNF-α stimulation. The numbers below the gel indicate the relative RPS3 signal intensity (normalized to PARP). D. Immunoprecipitation of RPS3 with C. rodentium NleH and EHEC NleH1 site-directed mutants. 293T cells were transfected with the indicated plasmids for 48 h and immunoprecipitated with an α-HA antibody. Immunoprecipitated samples were immunoblotted for RPS3 and HA. The top panel indicates immunoprecipitated RPS3 as a function of plasmid transfection (N.S. is a non-specific band, used for normalization of sample loading). The middle and bottom panels indicate RPS3 and HA abundance in the cell lysates, respectively.
(4.28 MB PDF)

Figure S6 Differential impact of NleH1 and NleH2 on NF-κB activity. A. Immunoblot analysis of RPS3 abundance after siRNA treatment. The numbers below the gel indicate the relative RPS3 signal intensity after normalization to tubulin. B. NF-κB activity (% activity compared to untreated samples) as a function of transfection with rps3 siRNA (open squares) and non-specific siRNA (closed squares). C. NF-κB activity as a function of transfection with RPS3-FLAG, in the presence of co-transfected HA (open circles), NleH1-HA (open squares), or NleH2-HA (closed squares). D. NF-κB activity as a function of transfection with HA (open circles), NleH1-HA (open squares), or NleH2-HA (closed squares).
(0.35 MB PDF)

Figure S7 Sequence alignment of NleH and OspG from STEC, *C. rodentium*, and *S. flexneri*. Homologous protein sequences were aligned by using ClustalW. Black box indicates the region of NleH1 interacting with RPS3. The symbols indicate the residues that were studied for their contribution to NleH1-mediated inhibition of RPS3/NF-κB activity (Δ, significant inhibition; *, partial inhibition; #, no inhibition). Coloring is used to highlight the sequence conservation of these residues among NleH proteins encoded by the indicated bacterial strains.
(0.27 MB PDF)
