# Supplementary material for: Bacterial Effector Binding to Ribosomal Protein S3 Subverts NF-κB Function
Source: PLoS Pathog. 2009 Dec 24;5(12):e1000708. doi: 10.1371/journal.ppat.1000708 (PMC2791202; doi:10.1371/journal.ppat.1000708)

A

```
NleH1:   1 MLSPYSVNLGCSWNSLTRNLTSPDNRVLSSVRDAAVHSDNGAQVKVGNRTYRVVATDNKF
NleH2:   1 MLSPSSINLGCSWNSLTRNLTSPDNRVLSSVRDAAVHSDSGTQVTVGNRTYRVVVTDNKF
           **** * ******************************** * ** ********* *****

        61 CVTRESHSGCFTNLLHRLGWPKGEISRKIEVMLNASPVSAAMERGIVHSNRPDLPPVDYA
        61 CVTRESHSGCFTNLLHRLGWPKGEISRKIEAMLNTSPVSTTIERGSVHSNRPDLPPVDYA
           ****************************** *** ****   *** **************

       121 PPELPSVDY----------NRLSVPGNVIGKGGNAVVYEDAEDATKVLKMFTTSQSNEEV
       121 QPELPPADYTQSELPRVSNNKSPVPGNVIGKGGNAVVYEDMEDTTKVLKMFTISQSHEEV
            ****  **          *   ***************** ** ******** *** ***

       171 TSEVRCFNQYYGAGSAEKIYGNNGDIIGIRMDKINGESLLNISSLPAQAEHAIYDMFDRL
       181 TSEVRCFNQYYGSGSAEKIYNDNGNVIGIRMNKINGESLLDIPSLPAQAEQAIYDMFDRL
           ************ *******  **  ***** ******** * ******* *********

       231 EQKGILFVDTTETNVLYDRAKNEFNPIDISSYNVSDRSWSESQIMQSYHGGKQDLISVVLSKI
       241 EKKGILFVDTTETNVLYDRMRNEFNPIDISSYNVSDISWSEHQVMQSYHGGKLDLISVVLSKI
           * *****************  *************** **** * ******** **********
```

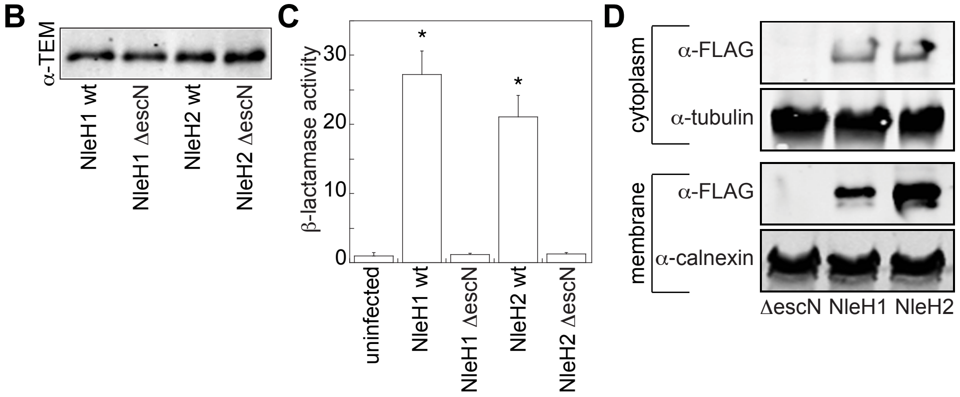

Supplement: Figure S1 — NleH amino acid sequences and T3SS-dependent translocation. A. E. coli EDL933 NleH1 (Z0989) and NleH2 (Z6021) amino acid sequences. Asterisks indicate identical residues. Amino acids differing between NleH1 and NleH2 are indicated in red. The lysine residue implicated in autophosphorylation activity is depicted in blue. B. Immunoblotting of bacterial lysates for NleH1- and NleH2-TEM expression in wild type (wt) or T3SS-deficient ΔescN EPEC (T3SS). Blots were probed with α-TEM antibody. C. β-lactamase activity (arbitrary units) in HeLa cells loaded with CCF2/AM substrate and infected for 4 h with wt or ΔescN EPEC (T3SS) strains expressing NleH1- or NleH2-TEM fusions. Asterisks indicate significantly different β-lactamase activity compared with uninfected samples (p<0.05, ANOVA). D. Immunoblot analysis of cytoplasmic and membrane HeLa cell fractions following infection with EPEC strains expressing NleH1- or NleH2-FLAG. Blots were probed with α-FLAG, α-tubulin, and α-calnexin antibodies. (0.46 MB PDF) [file ppat.1000708.s001.pdf]
