# Supplementary figures and images for: Bacterial Effector Binding to Ribosomal Protein S3 Subverts NF-κB Function
Source: PLoS Pathog. 2009 Dec 24;5(12):e1000708. doi: 10.1371/journal.ppat.1000708 (PMC2791202; doi:10.1371/journal.ppat.1000708)

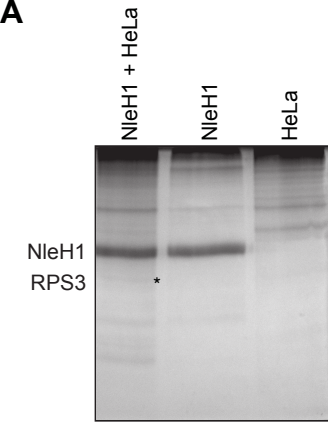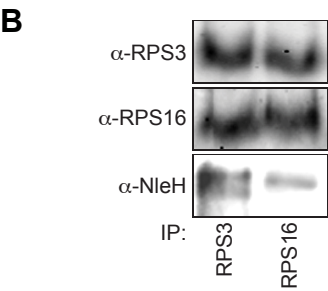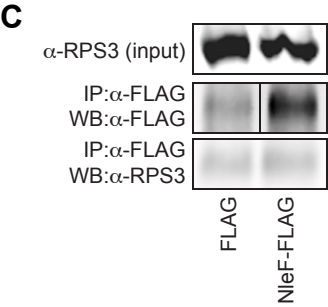

Supplement: Figure S2 — Binding specificity of NleH. A. Affinity enrichment of HeLa proteins with affinity for His-NleH1. HeLa cell lysates were incubated with purified His-NleH1 pre-bound to Ni-NTA agarose, eluted with imidazole, and analyzed by SDS-PAGE. Samples included HeLa lysate + His-NleH1 (lane 1), His-NleH1 (lane 2), and HeLa lysate (lane 3). Bands identified by mass spectrometry as NleH1 and RPS3 are indicated. B. NleH does not bind RPS16. HeLa cells were infected with EPEC expressing NleH1-FLAG and immunoprecipitated with α-RPS3 (left) or α-RPS16 (right) antibodies. The top and middle panels depict the abundance of RPS3 and RPS16 in the cell lysate, whereas the bottom panel depicts an α-FLAG immunoblot of the immunoprecipitated samples. Similar results were obtained with NleH2-FLAG. C. NleF does not bind RPS3. HeLa cells were infected with EPEC expressing FLAG (left) or NleF-FLAG (right) and immunoprecipitated with α-RPS3 antibody. The top panel depicts RPS3 in the cell lysates whereas the middle and bottom panels depict samples immunoprecipitated with α-FLAG antibody and subsequently immunoblotted for FLAG and RPS3, respectively. (1.56 MB PDF) [file ppat.1000708.s002.pdf]

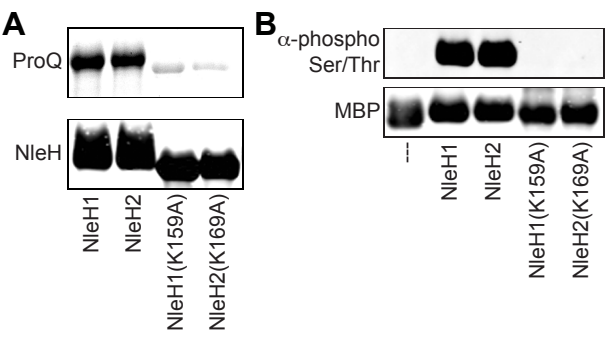

Supplement: Figure S3 — NleH1 and NleH2 are autophosphorylated Ser/Thr protein kinases. A. Autophosphorylation assay of His-NleH1 and NleH2, and site-directed mutants NleH1(K159A) and NleH2(K169A). Blots were stained with Pro-Q. B. Phosphorylation of myelin basic protein (MBP) by wild-type NleH1 and NleH2, but not the site-directed mutants NleH1(K159A) and NleH2(K169A). Blots were probed with α-His and α-phospho-Ser/Thr antibodies. (0.38 MB PDF) [file ppat.1000708.s003.pdf]

**A**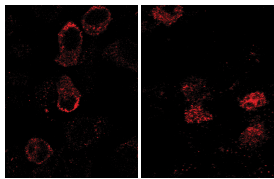**B**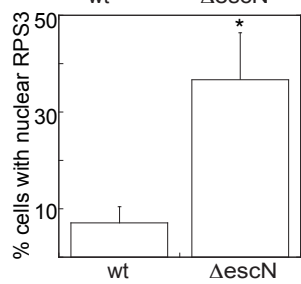

Supplement: Figure S4 — T3SS effector(s) inhibit RPS3 nuclear translocation. A. Immunofluorescence microscopy analysis of RPS3 nuclear abundance in HeLa cells infected with wild type (wt) or ΔescN EHEC. B. Quantification of the % of cells containing predominantly nuclear RPS3 (n = 100 cells). Asterisks indicate significantly different compared with wild-type infection (p<0.05, t-test). (0.95 MB PDF) [file ppat.1000708.s004.pdf]

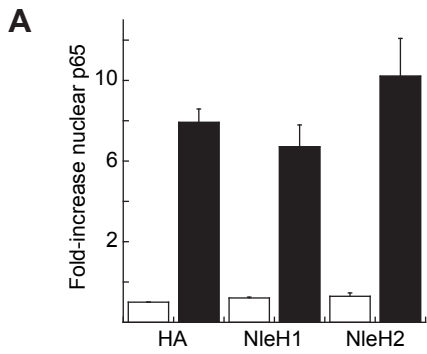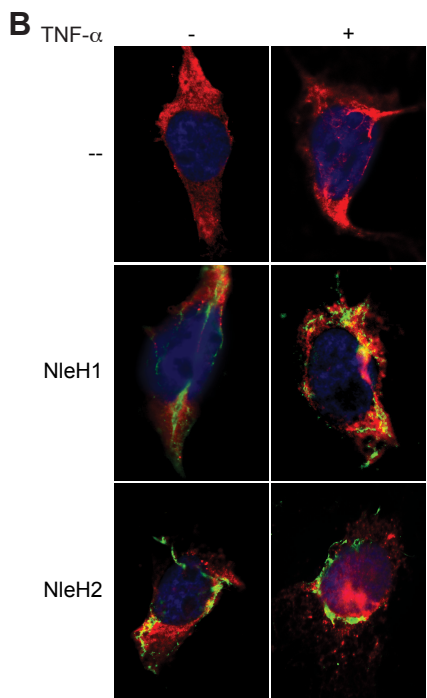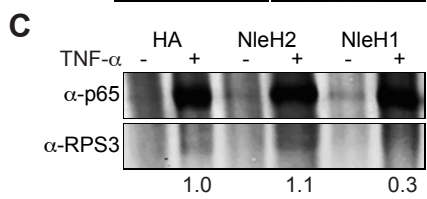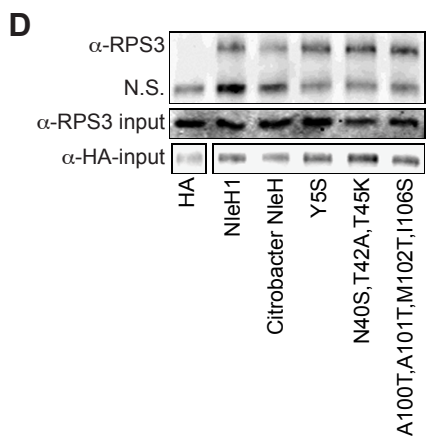

Supplement: Figure S5 — NleH1 reduces the nuclear abundance of RPS3. A. Quantification (n = 4) of the fold-increase in nuclear p65 as assessed from immunoblotting (depicted in Figure 5A), in the absence (open bars) or presence (black bars) of TNF-α stimulation. p65 signal intensity was normalized to PARP. B. Immunofluorescence microscopy analysis of NleH and RPS3 localization as a function of TNF-α stimulation. HeLa cells were infected for 3 h with EPEC strains expressing NleH1- or NleH2-FLAG, treated with TNF-α (100 ng/ml) for 1 h, and stained with DAPI (blue), a-FLAG (green), and a-RPS3 (red) monoclonal antibodies. C. Immunoprecipitation of nuclear extracts with α-p65 antibody. Immunoprecipitated samples were immunoblotted for p65 and RPS3 in samples transfected with the indicated plasmids, in the absence or presence of TNF-α stimulation. The numbers below the gel indicate the relative RPS3 signal intensity (normalized to PARP). D. Immunoprecipitation of RPS3 with C. rodentium NleH and EHEC NleH1 site-directed mutants. 293T cells were transfected with the indicated plasmids for 48 h and immunoprecipitated with an α-HA antibody. Immunoprecipitated samples were immunoblotted for RPS3 and HA. The top panel indicates immunoprecipitated RPS3 as a function of plasmid transfection (N.S. is a non-specific band, used for normalization of sample loading). The middle and bottom panels indicate RPS3 and HA abundance in the cell lysates, respectively. (4.28 MB PDF) [file ppat.1000708.s005.pdf]

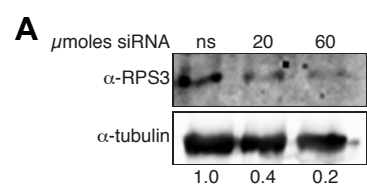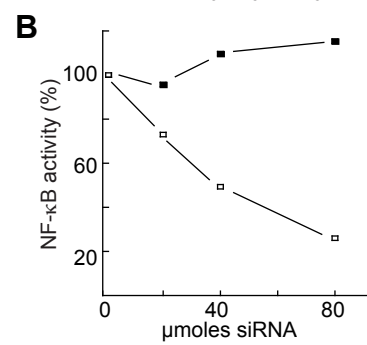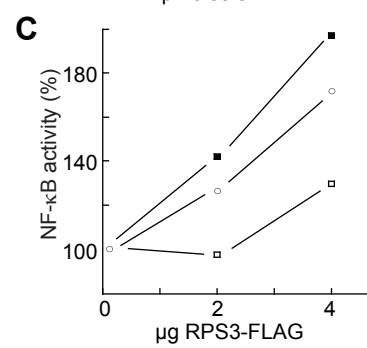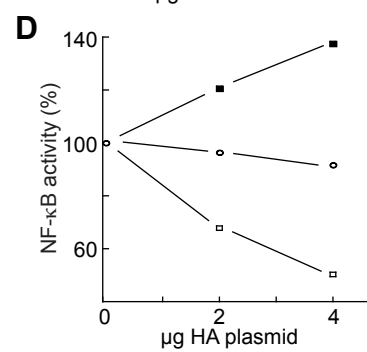

Supplement: Figure S6 — Differential impact of NleH1 and NleH2 on NF-κB activity. A. Immunoblot analysis of RPS3 abundance after siRNA treatment. The numbers below the gel indicate the relative RPS3 signal intensity after normalization to tubulin. B. NF-κB activity (% activity compared to untreated samples) as a function of transfection with rps3 siRNA (open squares) and non-specific siRNA (closed squares). C. NF-κB activity as a function of transfection with RPS3-FLAG, in the presence of co-transfected HA (open circles), NleH1-HA (open squares), or NleH2-HA (closed squares). D. NF-κB activity as a function of transfection with HA (open circles), NleH1-HA (open squares), or NleH2-HA (closed squares). (0.35 MB PDF) [file ppat.1000708.s006.pdf]
