# Supplementary material for: Bacterial Effector Binding to Ribosomal Protein S3 Subverts NF-κB Function
Source: PLoS Pathog. 2009 Dec 24;5(12):e1000708. doi: 10.1371/journal.ppat.1000708 (PMC2791202; doi:10.1371/journal.ppat.1000708)
Supplement: Figure S7 — Sequence alignment of NleH and OspG from STEC, C. rodentium, and S. flexneri. Homologous protein sequences were aligned by using ClustalW. Black box indicates the region of NleH1 interacting with RPS3. The symbols indicate the residues that were studied for their contribution to NleH1-mediated inhibition of RPS3/NF-κB activity (Δ, significant inhibition; *, partial inhibition; #, no inhibition). Coloring is used to highlight the sequence conservation of these residues among NleH proteins encoded by the indicated bacterial strains. (0.27 MB PDF) [file ppat.1000708.s007.pdf]

```
                       #                                      ^ ^  ^                                                                                ***   *
      NleH1 EDL933 MLSPYSVNLGCSWNSLTRNLTSPDNRVLSSVRDAAVHSDNGAQVKVGNRTYRVVATDNKFCVTRESHSGCFTNLLHRLGWPKGEISRKIEVMLNASPVSAAMERGIVHSNRPDLPPVDYAPPELPSVDY----------NRLSVPGN
    O157:NM-15842 MLSPYSVNLGCSWNSLTRNLTSPDNRVLSSVRDAAVHSDNGAQVKVGNRTYRVVATDNKFCVTRESHSGCFTNLLHRLGWPKGEISRKIEVMLNASPVSAAMERGIVHSNRPDLPPVDYAPPELPSVDY----------NRLSVPGN
 O119:H25-EC92-267 MLSPYSVNLGCSWNSLTRNLTSPDNRVLSSVRDAAVHSDNGAQVKVGNRTYRVVATDNKFCVTRESHSGCFTNLLHRLGWPKGEISRKIEVMLNASPVSAAMERGIVHSNRPDLPPVDYAPPELPSVDY----------NRLSVPGN
       NleH1 Sakai MLSPYSVNLGCSWNSLTRNLTSPDNRVLSSVRDAAVHSDNGAQVKVGNRTYRVVATDNKFCVTRESHSGCFTNLLHRLGWPKGEISRKIEVMLNASPVSAAMERGIVHSNRPDLPPVDYAPPELPSVDY----------NRLSVPGN
    O157:H7-278F1 MLSPYSVNLGCSWNSLTRNLTSPDNRVLSSVRDAAVHSDNGAQVKVGNRTYRVVATDNKFCVTRESHSGCFTNLLHRLGWPKGEISRKIEVMLNASPVSAAMERGIVHSNRPDLPPVDYAPPELPSVDY----------NRLSVPGN
      NleH2 EDL933 MLSPSSINLGCSWNSLTRNLTSPDNRVLSSVRDAAVHSDSGTQVTVGNRTYRVVVTDNKFCVTRESHSGCFTNLLHRLGWPKGEISRKIEAMLNTSPVSTTIERGSVHSNRPDLPPVDYAQPELPPADYTQSELPRVSNNKSPVPGN
       NleH2 Sakai MLSPSSINLGCSWNSLTRNLTSPDNRVLSSVRDAAVHSDSGTQVTVGNRTYRVVVTDNKFCVTRESHSGCFTNLLHRLGWPKGEISRKIEAMLNTSPVSTTIERGSVHSNRPDLPPVDYAQPELPPADYTQSELPRVSNNKSPVPGN
      C. rodentium MLSPAPVNLGCSWNSLTRNLTSPDNAVLSSVRDAAACSDNGTQVKVGNRIYRVVVTDNKFCVARENHSGCFTNMLHRLGWPKGEITRKIEAMLNTSPVNLAMERGSVHLKRPDLPPVDYMQPELPRVDY----------NKSPVPGN
          O111:H- MLSPSSVNLGCSWNSLTRNLTSPDSRILSSVRDAAASSDNGAQVKVGNRTYRVVVTDNKFCVTRESHSGCFTNLLHRLGWPKGEISRKIEVMLNSSPVNRAMERGAVHSNRPDLPPVDYAPPELPSVDY----------NSLPVPGN
          O26:H11 MLSPSSVNLGCSWNSLTRNLTSPDSRILSSVRDAAASSDNGAQVKVGNRTYRVVVTDNKFCVTRESHSGCFTNLLHRLGWPKGEISRKIEVMLNSSPVNRAMERGAVHSNRPDLPPVDYAPPELPSVDY----------NSLPVPGN
    O111:NM-R82F2 MLSPSSVNLGCSWNSLTRNLTSPDSRILSSVRDAAASSDNGAQVKVGNRTYRVVVTDNKFCVTRESHSGCFTNLLHRLGWPKGEISRKIEVMLNSSPVNRAMERGAVHSNRPDLPPVDYAPPELPSVDY----------NSLPVPGN
  O88:H25-EC94-453 MLSPSSVNLGCSWNSLTRNLTSPDSRILSSVRDAAASSDNGAQVKVGNRTYRVVVTDNKFCVTRESHSGCFTNLLHRLGWPKGEISRKIEVMLNSSPVNRAMERGAVHSNRPDLPPVDYAPPELPSVDY----------NSLPVPGN
  O69:H11-EC97-821 MLSPSSVNLGCSWNSLTRNLTSPDSRILSSVRDAAASSDNGAQVKVGNRTYRVVVTDNKFCVTRESHSGCFTNLLHRLGWPKGEISRKIEVMLNSSPVNRAMERGAVHSNRPDLPPVDYAPPELPSVDY----------NSLPVPGN
       11128 OspG ------------------------MKIISTVIQTPFPFEN-----------------------NNSHTGVVT----------------------------------------------------------------------EPILGK
 S. flexneri OspG ------------------------MKITSTIIQTPFPFEN-----------------------NNSHAGIVT----------------------------------------------------------------------EPILGK

      NleH1 EDL933 VIGKGGNAVVYEDAEDATKVLKMFTTSQSN-----EEVTSEVRCFNQYYGAGSAEKIYGNNGDIIGIRMDKINGESLLNIS--SLPAQAEHAIYDMFDRLEQKGILFVDTTETNVLYDRAKNEFNPIDISSYNVSDRSWSESQ---IMQSYHGGKQDLISVVLSKI-
    O157:NM-15842 VIGKGGNAVVYEDAEDATKVLKMFTTSQSN-----EEVTSEVRCFNQYYGAGSAEKIYGNNGDIIGIRMDKINGESLLNIS--SLPAQAEHAIYDMFDRLEQKGILFVDTTETNVLYDRAKNEFNPIDISSYNVSDRSW--------------------------
 O119:H25-EC92-267 VIGKGGNAVVYEDAEDATKVLKMFTTSQSN-----EEVTSEVRCFNQYYGAGSAEKIYGNNGDIIGIRMDKINGESLLNIS--SLPAQAEHAIYDMFDRLEQKGILFVDTTETNVLYDRAKNEFNPIDISSYNVSDRSWSESQ---IMQSYHGGKLDLISVV-----
       NleH1 Sakai VIGKGGNAVVYEDAEDATKVLKMFTTSQSN-----EEVTSEVRCFNQYYGAGSAEKIYGNNGDIIGIRMDKINGESLLNIS--SLPAQAEHAIYDMFDRLEQKGILFVDTTETNVLYDRAKNEFNPIDISSYNVSDRSWSESQ---IMQSYHGGKQDLISVVLSKI-
    O157:H7-278F1 VIGKGGNAVVYEDAEDATKVLKMFTTSQSN-----EEVTSEVRCFNQYYGAGSAEKIYGNNGDIIGIRMDKINGESLLNIS--SLPAQAEHAIYDMFDRLEQKGILFVDTTETNILYDRAKNEFNPIDISSYNVSDRSWSESQ---IMQSYHGGKQDLISVVLSK--
      NleH2 EDL933 VIGKGGNAVVYEDMEDTTKVLKMFTISQSH-----EEVTSEVRCFNQYYGSGSAEKIYNDNGNVIGIRMNKINGESLLDIP--SLPAQAEQAIYDMFDRLEKKGILFVDTTETNVLYDRMRNEFNPIDISSYNVSDISWSEHQ---VMQSYHGGKLDLISVVLSKI-
       NleH2 Sakai VIGKGGNAVVYEDMEDTTKVLKMFTISQSH-----EEVTSEVRCFNQYYGSGSAEKIYNDNGNVIGIRMNKINGESLLDIP--SLPAQAEQAIYDMFDRLEKKGILFVDTTETNVLYDRMRNEFNPIDISSYNVSDISWSEHQ---VMQSYHGGKLDLISVVLSKI-
      C. rodentium VIGKGGNAVVYEDMDDTTKVLKMFTTSQNP-----EEVTNEVSCFNQYYGSGSAEKIYDANGDIIGIRMNKINGESLFNIQ--SLPTQAEQAIYDMFDRLEQKRILFVDTTETNVLYDRVRNEFNPIDISSYNISDVSWREGQ---IMQSYHGGKQDLIRVVLSRI-
          O111:H- VIGKGGNAVVYEDAEDATKVLKMFTTSQSN-----EEVTNEVRCFNQYYGAGSAEKIYGDNGDIIGIRMDKINGESLLNIS--SLPAQAEHAIYDMFDRLEQKGILFIDTTETNVLYDRTRNEFNPIDISSYNISERSWSENQ---IMQSYHGGKQDLISVVLSKI-
          O26:H11 VIGKGGNAVVYEDAEDATKVLKMFTTSQSN-----EEVTNEVRCFNQYYGAGSAEKIYGDNGDIIGIRMDKINGESLLNIS--SLPAQAEHAIYDMFDRLEQKGILFIDTTETNVLYDRTRNEFNPIDISSYNISERSWSENQ---IMQSYHGGKQDLISVVLSKI-
    O111:NM-R82F2 VIGKGGNAVVYEDAEDATKVLKMFTTSQSN-----EEVTNEVRCFNQYYGAGSAEKIYGDNGDIIGIRMDKINGESLLNIS--SLPAQAEHAIYDMFDRLEQKGILFIDTTETNVLYDRTRNEFNPIDISSYNISERSWSENQ---IMQSYHGGKQDLISVVLSKI-
  O88:H25-EC94-453 VIGKGGNAVVYEDAEDATKVLKMFTTSQSN-----EEVTNEVRCFNQYYGAGSAEKIYGDNGDIIGIRMDKINGESLLNIS--SLPAQAEHAIYDMFDRLEQKGILFIDTTETNVLYDRTRNEFNPIDISSYNISERSWSENQ---IMQSYH--------------
  O69:H11-EC97-821 VIGKGGNAVVYEDAEDATKVLKMFTTSQSN-----EEVTNEVRCFNQYYGAGSAEKIYGDNGDIIGIRMDKINGESLLNIS--SLPAQAEHAIYDMFDRLEQKGILFIDTTETNVLYDRTRNEFNPIDISSYNISERSWSENQ---IMQSYHGGKQDLISV------
       11128 OspG LIGQGSTAEIFEDMNDSSALYKKYDLVGNQHNEVLEMARQESALFNTFYG-DDASVVIQYGGDV-YLRMLRVPGIPLSDIDTADIPDNLESLYLQLICKLNELSIIHYDLNTGNMLYDKESNSLFPIDFRNIYSEYYSATKNDKEIIDRRLQMRTNDFYSLLNRKYL
 S. flexneri OspG LIGQGSTAEIFEDVNDSSALYKKYDLIGNQYNEILEMAWQESELFNAFYG-DEASVVIQYGGDVY-LRMLRVPGTPLSDIDTADIPDNIESLYLQLICKLNELSIIHYDLNTGNMLYDKESESLFPIDFRNIYAEYYAATKKDKEIIDRRLQMRTNDFYSLLNRKYL
```
